# *ASXL1* truncating variants in BOS and myeloid leukemia drive shared disruption of Wnt-signaling pathways but have differential isoform usage of RUNX3

**DOI:** 10.1186/s12920-024-02039-7

**Published:** 2024-11-29

**Authors:** Isabella Lin, Zain Awamleh, Mili Sinvhal, Andrew Wan, Leroy Bondhus, Angela Wei, Bianca E. Russell, Rosanna Weksberg, Valerie A. Arboleda

**Affiliations:** 1 Department of Pathology and Laboratory Medicine, David Geffen School of Medicine, UCLA, Los Angeles, CA USA; 2 Department of Computational Medicine, David Geffen School of Medicine, UCLA, Los Angeles, CA USA; 3 Department of Human Genetics, David Geffen School of Medicine, UCLA, Los Angeles, CA USA; 4 Department of Genetics and Genome Biology, The Hospital for Sick Children, Toronto, ON Canada; 5 Interdepartmental Bioinformatics Program, UCLA, Los Angeles, CA USA; 6 Department of Human Genetics, Division of Clinical Genetics, UCLA, Los Angeles, CA USA; 7 Department of Pediatrics, Division of Clinical & Metabolic Genetics, The Hospital for Sick Children, Toronto, ON Canada; 8 Institute of Medical Sciences, Department of Molecular Genetics, University of Toronto, Toronto, ON Canada; 9 Molecular Biology institute, UCLA, Los Angeles, CA USA; 10 Jonsson Comprehensive Cancer Center, UCLA, Los Angeles, CA USA

**Keywords:** ASXL1, Acute myeloid leukemia, Bohring-Opitz syndrome, Transcriptomics, Epigenetics, Multi-omics, DNA methylation, RNA-sequencing

## Abstract

**Background:**

Rare variants in epigenes (a.k.a. chromatin modifiers), a class of genes that control epigenetic regulation, are commonly identified in both pediatric neurodevelopmental syndromes and as somatic variants in cancer. However, little is known about the extent of the shared disruption of signaling pathways by the same epigene across different diseases. To address this, we study an epigene, Additional Sex Combs-like 1 (*ASXL1*), where truncating heterozygous variants cause Bohring-Opitz syndrome (BOS, OMIM #605039), a germline neurodevelopmental disorder, while somatic variants are driver events in acute myeloid leukemia (AML). No BOS patients have been reported to have AML.

**Methods:**

This study explores common pathways dysregulated by *ASXL1* variants in patients with BOS and AML. We analyzed whole blood transcriptomic and DNA methylation data from patients with BOS and AML with *ASXL1*-variant (AML-*ASXL1*) and examined differential exon usage and cell proportions.

**Results:**

Our analyses identified common molecular signatures between BOS and AML-*ASXL1* and highlighted key biomarkers, including *VANGL2*, *GRIK5* and *GREM2*, that are dysregulated across samples with *ASXL1* variants, regardless of disease type. Notably, our data revealed significant de-repression of posterior homeobox A (*HOXA*) genes and upregulation of Wnt-signaling and hematopoietic regulator *HOXB4*. While we discovered many shared epigenetic and transcriptomic features, we also identified differential splice isoforms in *RUNX3* where the long isoform, p46, is preferentially expressed in BOS, while the shorter p44 isoform is expressed in AML-*ASXL1.*

**Conclusion:**

Our findings highlight the strong effects of *ASXL1* variants that supersede cell-type and even disease states. This is the first direct comparison of transcriptomic and methylation profiles driven by pathogenic variants in a chromatin modifier gene in distinct diseases. Similar to RASopathies, in which pathogenic variants in many genes lead to overlapping phenotypes that can be treated by inhibiting a common pathway, our data identifies common pathways for *ASXL1* variants that can be targeted for both disease states. Comparative approaches of high-penetrance genetic variants across cell types and disease states can identify targetable pathways to treat multiple diseases. Finally, our work highlights the connections of epigenes, such as *ASXL1*, to an underlying stem-cell state in both early development and in malignancy.

**Supplementary Information:**

The online version contains supplementary material available at 10.1186/s12920-024-02039-7.

## Key points

• ASXL1-driven transcriptomic and DNA methylation dysregulation highlight upregulation of Wnt-signaling pathways and aberrant posterior *HOX* gene regulation.

• Differential RUNX3 isoform usage between BOS and AML-*ASXL1* distinguishes between normal, in BOS, and abnormal, in AML, hematopoiesis.

• The ASXL1-centric approach demonstrates that *ASXL1* variants affect some common pathways and mechanisms and highlight the potential for common therapeutic targets.

**Supplementary Information:**

The online version contains supplementary material available at 10.1186/s12920-024-02039-7.

## Introduction

Bohring-Opitz syndrome (BOS, OMIM#605309) [1] and acute myeloid leukemia (AML) are two diseases with distinct clinical presentations; BOS is a pediatric, neurodevelopmental disorder caused by germline variants in the additional sex combs-like 1 (*ASXL1*) gene [2, 3] while AML is a hematologic malignancy derived from myeloid progenitor and hematopoietic stem cells (HSCs) in the bone marrow in which somatic *ASXL1* variants are a common driver variant. These two diseases are driven by the same pathogenic, protein-truncating variants in *ASXL1*. Early developmental disorders and malignancies share a common central cell type: the stem cell, which drives the ensuing disease and highlights potential common molecular mechanisms and cellular targets across clinically distinct disorders.

BOS is characterized by severe to profound intellectual disability, distinctive facial features, and congenital anomalies that affect multiple organ systems [2, 3]. Patients with BOS are at increased risk of developing Wilms tumor and hepatoblastoma [3, 4], rare embryonic kidney and liver tumors that occur in children, yet there are no reported cases of myeloid leukemias, even into the third decade of life [2, 3]. In contrast, myeloid malignancies– including chronic myelomonocytic leukemia (CMML), myelodysplastic syndromes (MDS), myeloproliferative neoplasms, and both secondary and *de novo* AML [5–8], exhibit a diverse genetic mutational landscape of which somatic variants of *ASXL1* are frequently observed. The presence of *ASXL1* variants in AML (AML-ASXL1) are associated with poor overall survival and therapeutic outcome [5]. However, the role of *ASXL1* variants across these distinct disorders has not been compared.

Over fifty years ago, Dr. Beatrice Mintz proposed a connection between development and cancer, positing that genetic anomalies in stem cells can lead to cancer by inducing a reversion to an undifferentiated state [9–11]. The class of genes that regulate the epigenome, termed epigenes [12–14], direct the epigenome structure and play a pivotal role in stem cell differentiation [15, 16] and cancer pathogenesis [17] through control of RNA expression and complex biological signaling [18]. Variants in epigenes dysregulate developmental programs, resulting in structural anomalies or, in somatic variants, reverting cells to an early state with malignant potential [19]. Although the dual presence of epigene variants in both human development and cancer has been documented across numerous studies [20], the specific pathogenic mechanisms driven by variants in the same gene across distinct diseases remain largely unexplored.

*ASXL1* encodes *Additional sex combs like 1*, which functions as a transcriptional regulator and chromatin remodeler within three polycomb repressive complexes (PRC): PRC1, PRC2, and Polycomb Repressive Deubiquitinase (PR-DUB) complex [21, 22]. Despite the crucial role of ASXL1 in development, the factors that control ASXL1 and its interactions with one or more of these complexes remain largely unknown. Previous studies have demonstrated that ASXL1 modulates the balance between the proliferation and differentiation of stem progenitor cell populations. Variants in ASXL1 can disrupt this equilibrium, favoring a stem-cell identity over differentiation in both BOS and myeloid leukemias [23, 24]. In the context of myeloid leukemia, mutations in *ASXL1* have been shown to lead to the loss of *ASXL1* expression and a consequent reduction of PRC2-mediated histone 3 lysine 27 tri-methylation (H3K27me3), a histone modification associated with gene repression [25].

ASXL1 associates with the PRC2 complex to mediate several downstream events. ASXL1 interacts with PRC2 core components - such as enhancer of zeste homolog 2 (EZH2), a key PRC2 protein that interacts with DNA methyltransferases to modulate DNA methylation (DNAm) while catalyzing specific histone methylation including H3K27me3 [25, 26]. EZH2 is typically enriched at the posterior end of the homeobox A (HOXA) cluster locus, where PRC2 mediates transcriptional repression. The loss of ASXL1 results in reduced EZH2 enrichment at this locus, indicating that *ASXL1* plays an essential role in EZH2-mediated repression of the *HOXA* locus [25]. These interactions highlight how disruptions in ASXL1 can significantly alter both the gene expression and DNA methylation landscape.

Furthermore, overexpression of ASXL1 variants and mouse knockouts have linked ASXL1 to the regulation of splicing [24, 27] suggesting yet another role of *ASXL1* during development. Alternative splicing is thought to play a key regulatory role in modulating transitions between stem cell differentiation, proliferation and tissue development. Our previous work studying BOS patient-derived samples, which harbor germline *ASXL1* variants, found that many epigenetic and transcriptomic changes are cell-type specific, but there are clear disruptions that are shared across *ASXL1*-mutated cells, such as dysregulation of the Wnt-signaling pathways [28].

In this study, we prioritized a gene-centric approach, with the hypothesis that *ASXL1* variants disrupt the same core pathways regardless of individual genetic background and clinical disease. While these, as well as sex and age and other factors, can contribute to the variance observed in transcriptome and epigenomic data, ASXL1 drives a clear and shared genetic dysregulation across both diseases. Our recent study across BOS patient-derived blood and fibroblasts identified epigenomic and transcriptomic changes associated with *ASXL1* variants across tissues, such as the upregulation of Van Gogh-like 2 (VANGL2) [28], a gene associated with non-canonical Wnt-signaling and migration. By integrating distinct disease datasets that share a common pathogenic variant, we aim to pinpoint key molecular events driven by *ASXL1* variants and understand how these drive distinct clinical manifestations.

This study explores the landscape of *ASXL1* variants driving two distinct diseases - BOS and AML. By employing a comprehensive integrative approach for RNA-sequencing (RNA-seq), DNAm, and exon usage analyses, we found that BOS and AML-*ASXL1* patient-derived samples shared an upregulation of Wnt-signaling and DNAm mediated de-repression of specific *HOX* genes - *HOXB4* and *HOXA11*. However, there remain differences in isoform expression analysis, with distinct *RUNX3* isoforms expressed in blood from BOS compared to AML-*ASXL1* patient samples. This is the first study to explicitly link and compare the shared epigenetic and transcriptomic changes initiated by *ASXL1* variants and highlights potential therapeutic biomarkers.

## Samples and methods

### Selection and characterization of BOS and AML samples

Our study included data from patient cohorts with Bohring-Opitz syndrome (BOS) and acute myeloid leukemia (AML). Specifically, we leveraged samples from patients with AML harboring *ASXL1* variants (AML-*ASXL1*, Table 1). Informed consent was obtained from all research participants according to the protocol approved by the Hospital for Sick Children (REB#1000038847) and UCLA (IRB#11-001087). Illumina 450K DNA methylation (DNAm) data was acquired for six AML samples from The Cancer Genome Atlas (TCGA) program [29], available on the Genomic Data Commons (GDC) repository [30]. Among these, three samples had somatic *ASXL1* variants, serving as the AML-*ASXL1* cohort, and three had somatic variants in other genes, serving as AML controls. Transcriptomic data for AML-*ASXL1* bone marrow samples (*n* = 28, samples with evidence of *ASXL1* variant in both DNA and RNA) were sourced from the Beat AML cohort (phs001657.v3.p1) [31] and AML-*ASXL1* blood samples (*n* = 6) from TCGA, with non-AML blood controls (*n* = 60) from the Genotype-Tissue Expression (GTEx) Portal, and bone marrow controls (*n* = 8) from the publicly available dataset (GSE120444) [32] (Table 1 and Table S1). Since AML-*ASXL1* samples can have multiple variants we listed all other variants in Table 1. Additionally, blood samples for RNA-seq and DNAm data from BOS patients (*n* = 8 RNA-seq, *n* = 8 DNAm) and healthy blood controls from REACH biobank (*n* = 11 RNA-seq, *n* = 26 DNAm) were collected from our previous studies and are publicly available at GSE230685 and GSE230696 [28, 33]. A subset of patient-derived blood (*n* = 8 BOS, *n* = 10 healthy controls, *n* = 4 AML-*ASXL1*, *n* = 6 AML controls) RNA-seq data was used to conduct differential exon usage (DEU) and isoform expression analysis (Table 1).

**Table 1:**
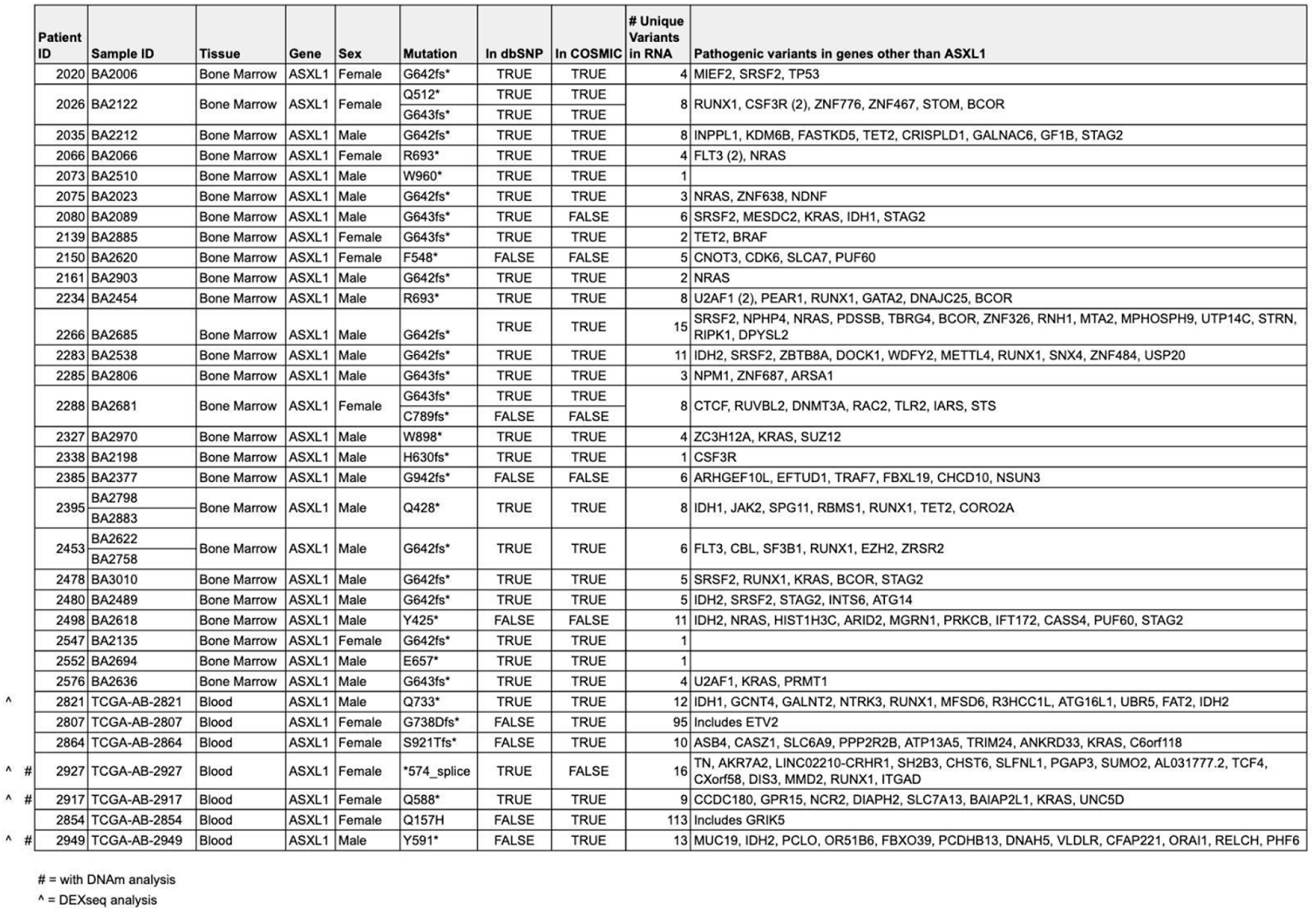
Demographic, clinical, and genetic characteristics of AML-*ASXL1* patients

This table presents a comprehensive overview of the demographic, clinical, and genetic characteristics of acute myeloid leukemia (AML) patients with ASXL1 variants (AML-*ASXL1*) used in this study. The data provided includes tissue type, gender, *ASXL1* variant, and other relevant clinical parameters. Detailed genetic information for AML-*ASXL1* samples at the *ASXL1* variant sites are provided with alternate allele read, total read, cancer allele frequency, ExAC frequency, and variant registration in dbSNP or COSMIC databases. Other unique pathogenic variants found in the samples are also provided

# indicates samples also used for DNAm analysis

^ indicates samples also used for DEXseq analysis

### Sample collection, processing, and sequencing

For DNA and RNA extraction, peripheral blood samples were processed using standardized protocols [28, 33], with EDTA tubes for DNA and PAXgene Blood Tubes (BDBiosciences, 762165) for RNA. Genomic DNA was extracted from peripheral blood and bisulfite converted using the EpiTect Bisulfite Kit (EpiTect PLUS Bisulfite Kit, Qiagen, #59124) before being hybridized to the Illumina Infinium Human Methylation EPIC BeadChip following established methods [33]. Cases and controls were randomly assigned a chip position and run in a single batch to reduce batch effects. REACH Biobank RNA-seq libraries were prepared using TruSeq Stranded TotalRNA Library Prep Gold (Illumina, #20020599) with QiaSelect rRNA and globin depletion (Qiagen, #334376 #334386) following established methods [28]. Pooled libraries were sequenced to 40 million reads per sample on a NovaSeq6000, and are publicly available datasets deposited at GSE230685 and GSE230686 [28]. TCGA samples and samples obtained from GSE120444 [32] were also prepared with TruSeq Stranded Total RNA Library Prep Kit (Illumina). GTEx RNA-seq libraries were prepared with TruSeq RNA Library Prep Kit (Illumina). BEAT-AML samples were prepared using Agilent SureSelect Strand-Specific RNA Library Preparation Kit for polyA(+) RNA [31].

### RNA-seq and DNA methylation analysis of BOS and AML samples

#### Preprocessing and quality control of RNA-seq and DNA methylation data

RNA-seq data was processed using our established pipeline [28]. Briefly, reads were mapped to hg38 using STAR 2.7.0e [34], gene counts were generated using featureCounts 1.6.5 [35] and used a gene set of GenCode hg38 annotation v31, composed of 60,662 genes and differential expression adjusted p-value (p_adj_) and log2 fold change (log2FC) were quantified using DESeq2 v1.24.0 [36], correcting for sex and tissue (Table 1 and Table S1).

DNAm data underwent processing through our previously published pipeline [33]. Briefly, the minfi Bioconductor package in R was used to preprocess data including quality control, Illumina normalization and background extraction, followed by identification and filtering of highly differentially methylated sites (|delta beta (Δβ)| > 5%). Significant CpG sites were identified with FDR < 0.05. Principal component analysis (PCA) and hierarchical clustering utilized the BOS DNAm episignature sites (413 CpG sites) [33].

### Integration of RNA-seq and DNA methylation data

Integration of RNA-seq and DNAm data utilized beta values for DNAm [37] and transcript per million (TPM) values for RNA-seq [38], enabling the identification of shared and distinct molecular signatures across the conditions studied.

### DEXSeq analysis of bulk RNA-seq datasets

The Bioconductor R package DEXSeq v1.50.0 [39] was used to quantify DEU from RNA-seq data. The reads mapping to a single exon bin were normalized against those mapping to all exon bins within the same gene, where an exon bin is a whole exon or part of an exon that arises when an exonic region occurs in different transcripts with varying boundaries. These exon bins were then compared across conditions to test for DEU. Preprocessing was done using two Python scripts built into the DEXSeq package, with the Python package HTSeq. Samtools v1.20 was used to convert BAM files to SAM files. P-values were adjusted for multiple testing by DEXSeq using the Benjamini-Hochberg method. Significant exon bins with DEU were identified with padj < 0.05.

### Pathway mapping using KEGG mapper

KEGG Mapper was used to model and visualize the set of differentially expressed genes (DEGs) in the context of biological pathways and molecular networks [40, 41].

### Cell type deconvolution using CIBERSORTx

CIBERSORTx is an online bioinformatics tool that assesses cell type-specific gene expression profiles and cellular composition from RNA-seq data [42]. Bulk RNA-seq datasets were compared against the LM22 deconvolution signature matrix containing marker gene profiles to impute cell type proportions and cell expression profiles [43]. LM22 is a signature matrix file consisting of 547 genes that distinguishes between 22 mature human hematopoietic populations from peripheral blood [44].

## Results

### Study design and genetic landscape of *ASXL1* variants

**Fig. 1:**
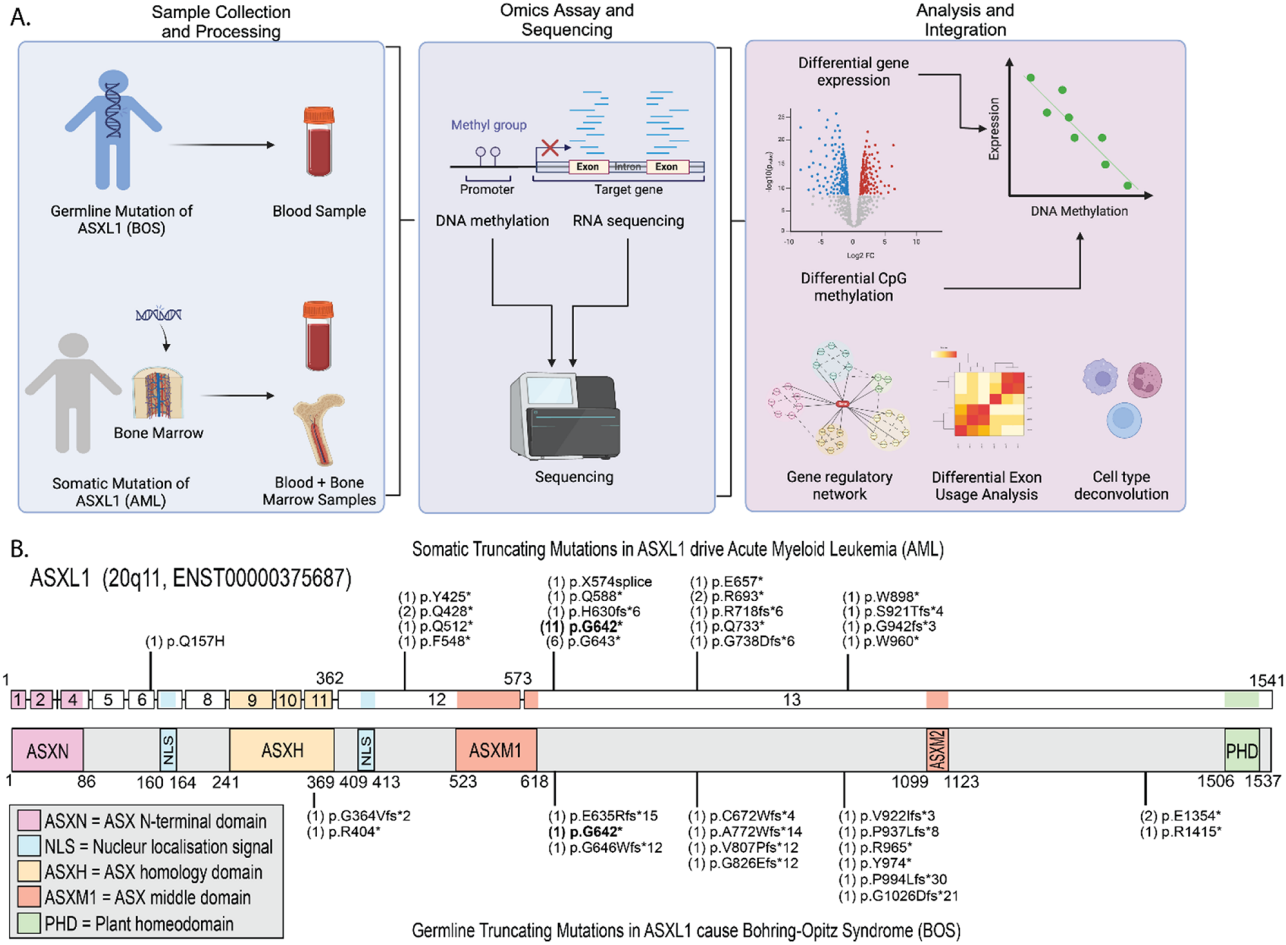
Study design and genetic landscape of *ASXL1* variants (**A**) Detailed workflow from the collection of patient samples through to the generation and analysis of RNA-sequencing (RNA-seq) and DNA methylation (DNAm) data. Blood samples were collected from Bohring-Opitz syndrome (BOS) patients, and control individuals, and RNA-seq and DNAm were conducted. We compared our BOS samples with RNA-seq and DNAm data from the BEAT AML and The Cancer Genome ATLAS data of in blood and bone marrow from acute myeloid leukemia with *ASXL1* variants (AML-*ASXL1*) or without *ASXL1* variants (AML), RNA-seq and DNAm analysis was conducted to examine differential gene expression, differential CpG methylation, dysregulated gene regulatory networks, differential exon usage, and cell type deconvolution. (**B**) The *ASXL1* gene, on chromosome 20q11, is illustrated, highlighting gene domains and the loci of germline variants that cause BOS (below the gene) and somatic variants that drive AML-*ASXL1* (above the gene) in this study. Common variant sites (bold font), illustrate the genetic intersections between BOS and AML-*ASXL1*, and the bracketed numbers preceding the variant annotation indicates the number of samples in this study with that variant. The majority of variants are in the last two exons of the *ASXL1* gene

To compare the epigenetic and transcriptomics effects of ASXL1 variants, we collected data from patients with BOS and AML with *ASXL1* variants (AML-*ASXL1*). For BOS patients and matched controls, patient blood samples were collected and RNA-sequencing (RNA-seq) and DNA methylation (DNAm) analyses were performed (Fig. 1A). For comparison across disease-states, we also collected RNA-seq and DNAm data from AML-*ASXL1* patients and tissue-matched controls from dbGAP repositories [31]. We re-processed all samples through our RNA-seq and DNAm pipeline to minimize analytical batch effects (Methods).

The *ASXL1* gene is made up of 13 exons and encodes a protein that spans 1541 amino acids. The majority of pathogenic variants in this study disrupt the latter half of the protein-coding region encoded by the last two exons of *ASXL1*. Figure 1B illustrates the germline BOS variants and somatic AML-*ASXL1* variants across the *ASXL1* gene in this study. Common variant sites between the two disorders are bolded. Variant details for the AML-*ASXL1* blood and bone marrow samples in this study are provided (Table 1). No significant differences were identified between BOS and AML-*ASXL1* variant allele frequency (VAF) for the pathogenic variant/s in *ASXL1*. AML*-ASXL1* variants had a slightly lower average VAF compared with BOS due to the fact that AML samples are heterogeneous, harboring both leukemia cells with *ASXL1* variants and non-leukemia cells which decreases the proportion of reads mapping to the *ASXL1* variant.

### *ASXL1* variants drive transcriptomic dysregulations across blood and bone marrow in AML

We next asked whether the pathogenic *ASXL1* variants might drive differentially expressed genes (DEGs) in AML samples. DEG analysis was conducted for AML-*ASXL1* bone marrow (*n* = 26), and AML-*ASXL1* blood samples from TCGA (*n* = 6), as well as tissue-matched healthy controls of blood from the GTEx portal (*n* = 60), and bone marrow samples from publicly available dataset GSE120444 (*n* = 8) samples.

To assess the role of tissue specificity on gene expression, we analyzed the AML-*ASXL1* data with the blood and bone marrow combined as well as independently with respective matched controls. Our principal components analysis showed that tissue type explained 17% of the variance along PC1, while the presence of an *ASXL1* variant explained 11% along PC2 (Figure S1A). In the subsets analyzed within the same tissue types, *ASXL1* variant status drove 34% variance in blood and 64% variance in bone marrow samples. *DESeq2* analysis was conducted to identify DEGs and these were visualized using unsupervised clustering (Figure S1B). This showed stronger clustering of samples by condition (*ASXL1* variant status) than by tissue type (blood or bone marrow).

*DESeq2* analysis identified 9527 DEGs, adjusted for tissue type, of which 7889/9527 met a log2 fold change (log2FC) cutoff of |log2FC| ≥ 0.58. Of these, 4610/7889 were upregulated (58.44%, log2FC > 0) and 3279/7889 were downregulated (41.56%, log2FC < 0) in the AML-*ASXL1* samples (Figure S1C). The 15 most upregulated and 15 most downregulated DEGs with the largest absolute log2FC are shown in Table S2. Using clusterProfiler, we conducted gene ontology analyses on the set of DEGs to identify dysregulated biological processes. We identified consistent dysregulation of epigenetic functions (p_adj_=6.51E-06), including histone modification (p_adj_=1.40E-05), chromatin modification (p_adj_=2.35E-05), DNA replication (p_adj_=1.30E-05) and conformational change (p_adj_=7.63E-05), and immune activation (p_adj_=1.30E-03) (Figure S1D).

### *ASXL1* variants in AML-*ASXL1* and BOS drive shared transcriptomic dysregulations

To identify DEGs that are common across different disease-types driven by *ASXL1* variants, we integrated these AML-*ASXL1* DEGs (AML-*ASXL1* compared to tissue-matched controls) with the 2118 significant DEGs previously identified in BOS blood samples (BOS *n* = 8 compared to healthy controls *n* = 11) [28].

**Fig. 2:**
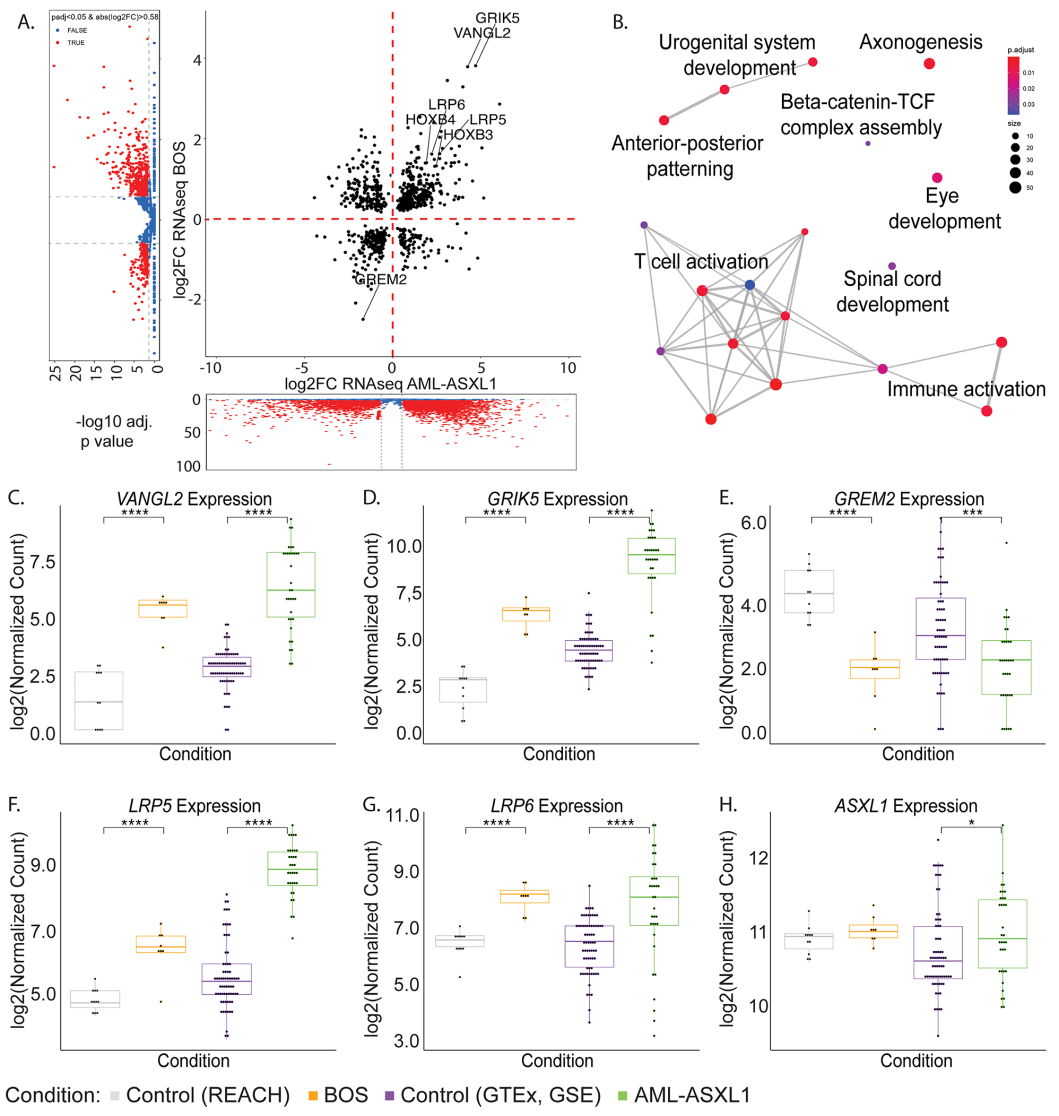
Transcriptomic alterations driven by *ASXL1* variants in BOS and AML-*ASXL1* highlight key biomarkers and Wnt signaling dysregulation. (**A**) Fold change integration plot of all 843 genes that were significantly differentially expressed genes (DEGs, p_adj_ < 0.05) in both AML-*ASXL1* and BOS compared to their respective controls. (**B**) Gene ontology of all common DEGs in AML-*ASXL1* and BOS revealed enrichment in T cell activation, anterior/posterior pattern specification, urogenital system development, axonogenesis, and beta-catenin-TCF complex assembly, among other pathways. Log2 normalized transcript expression analysis of BOS and AML-*ASXL1* samples and their respective controls identified key biomarkers and showed significant (**C**) upregulation in *VANGL2* (**D**) upregulation in *GRIK5*, and (**E**) downregulation in *GREM2.* Wnt signaling co-receptors (**F**) *LRP5* and (**G**) *LRP6* were also significantly upregulated in BOS and AML-*ASXL1* compared to controls. (**H**) *ASXL1* was significantly upregulated in AML-*ASXL1* but not in BOS. ns or no stars denote *p*-value > 0.05, * denote *p*-value ≤ 0.05, ** denote *p*-value < 0.01, *** denote *p*-value < 0.001, **** denote *p*-value < 0.0001

We found that *ASXL1* variants, regardless of disease and germline or somatic status, drove common transcriptional changes. We identified 843 common DEGs between the AML-*ASXL1* and BOS datasets, of which 566/843 (67.14%) DEGs were dysregulated in the same direction - either upregulated in both datasets, or downregulated in both datasets (Fig. 2A). We plotted fold change for DEGs from the transcriptomic analyses with AML-*ASXL1* compared to their respective controls on the x-axis, and BOS compared to their respective controls on the y-axis. This highlighted 388/843 (46.03%) DEGs with a large effect size, with an absolute fold change greater than 1.5 in both BOS and AML-*ASXL1*. Of these DEGs, 246/388 (63.40%) were dysregulated in the same direction. The 50 DEGs with the largest effect size in BOS are listed in Table 2, with respective log_2_FC and p_adj_ values.

**Table 2:**
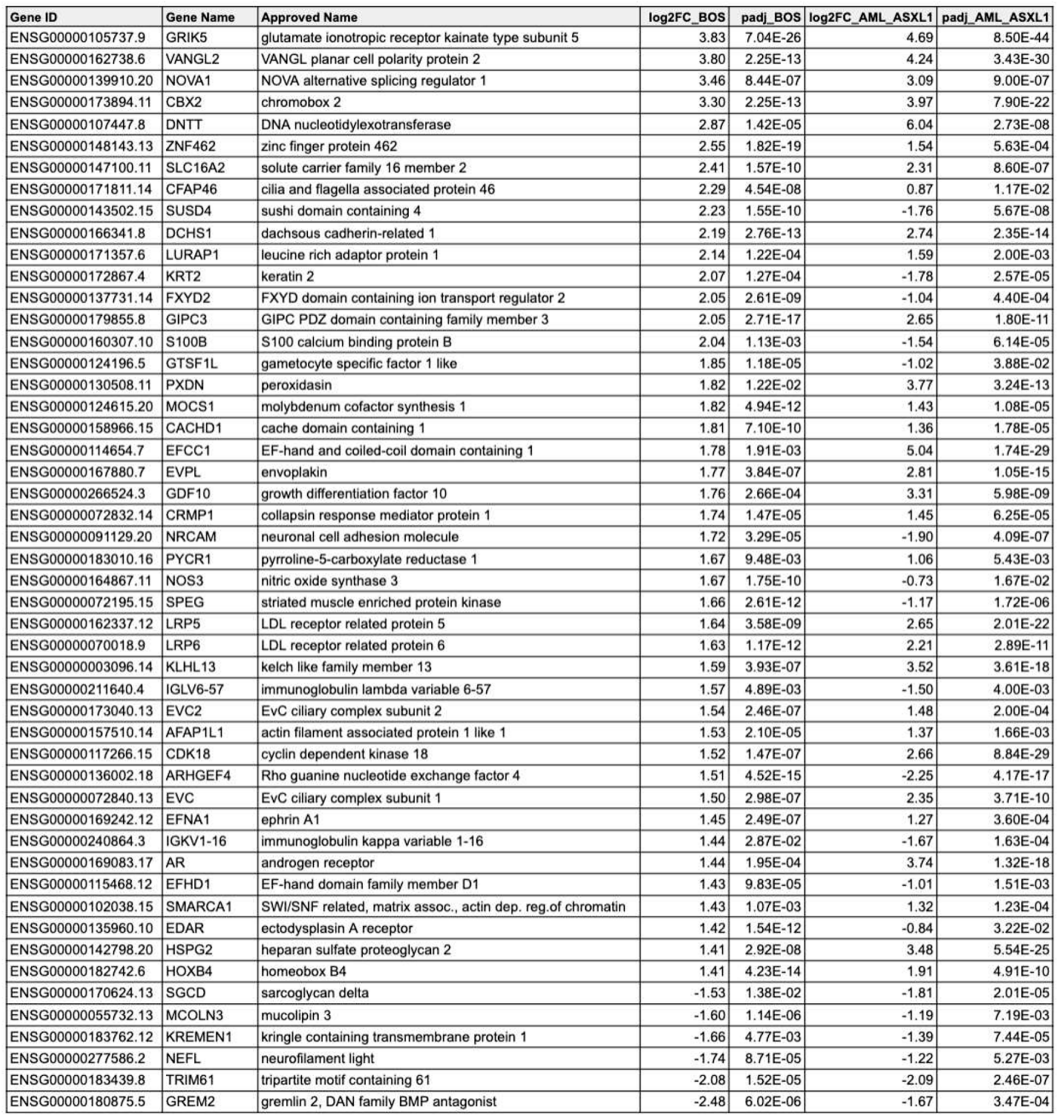
Comparative differentially expressed genes between AML-*ASXL1* (blood + bone marrow) and BOS RNA-seq analysis

Integration of differential gene expression analyses from AML-*ASXL1* blood and bone marrow samples and Bohring-Opitz syndrome (BOS) blood. This comparison displays the 50 most differentially expressed genes (DEGs), adjusted for tissue and sex, and includes gene ID, log_2_ fold changes (log_2_FC) for both datasets, adjusted p-values, and gene names

Gene ontology analyses demonstrated enrichment in biological processes such as T-cell activation (GO:0042110, p_adj_=4.35E-06), axonogenesis (GO:007409, p_adj_=4.52E-03), and anterior/posterior pattern specification (GO:009952, p_adj_=5.61E-03) (Table 3). While the common DEGs between the AML-*ASXL1* and BOS datasets identified T-cell activation and T-cell differentiation among other T-cell functions as significantly enriched pathways, further analysis identified that the DEGs driving these pathways were upregulated in BOS and downregulated in AML-*ASXL1*, which is consistent with differential T-cell compositions between blood and bone marrow; T-cells comprise approximately 6% of lymphocytes in the bone marrow and approximately 62% in peripheral blood [45]. BOS samples were derived only from blood samples while AML-ASXL1 samples included both blood and bone marrow samples. On the other hand, dysregulation of anterior/posterior pattern specification was driven by upregulation of genes in both BOS and AML-*ASXL1*.

**Table 3:**
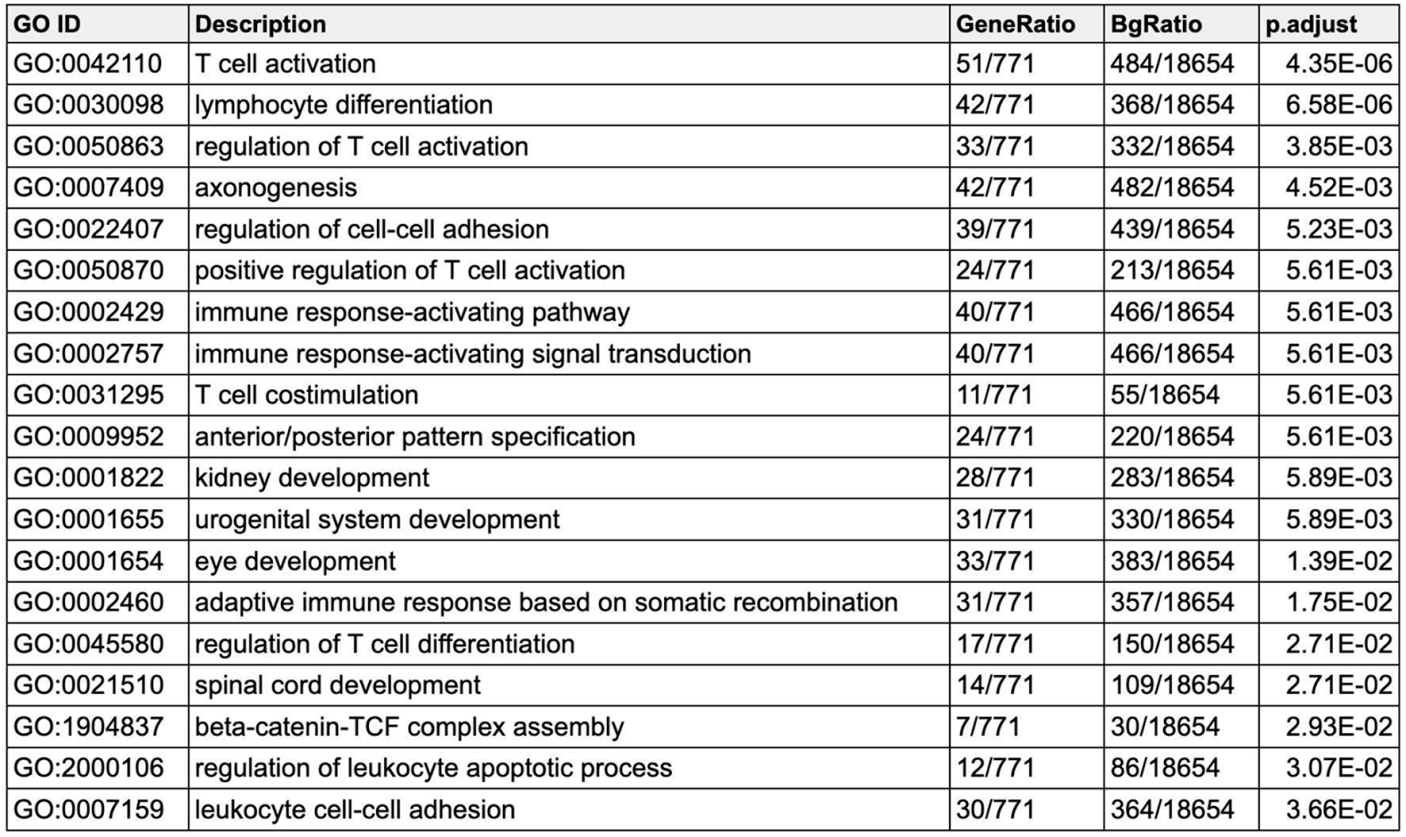
Gene ontology analysis for common DEGs in AML-*ASXL1* (blood + bone marrow) and BOS

Gene ontology (GO) analysis for the common differentially expressed genes (DEGs) identified in both AML-*ASXL1* (blood + bone marrow samples) and BOS (blood) analyses, adjusted for tissue and sex. This table lists the top 20 GO terms enriched among the commonly dysregulated genes, GO ID, description, gene ratio, and adjusted p-value

### Wnt-signaling pathways are dysregulated across BOS and AML-*ASXL1* samples

Of note, the genes associated with beta-catenin-TCF complex assembly, a key component of the canonical Wnt signaling pathway, were significantly dysregulated (p_adj_=2.71E-02) in the DEGs across both datasets (Fig. 2B). We previously identified that the canonical Wnt signaling pathway was aberrantly upregulated in BOS patient-derived samples [28]. We further analyzed this dysregulation of Wnt signaling through comprehensive gene ontology network analysis [46, 47]. We first depicted the consensus of DEGs against the KEGG pathway hsa05200 which represents the kernel regulatory factors that contribute to the initiation and progression of pan-cancer. This identified the KEGG pathway hsa04310, representing Wnt signaling pathway, as one of the most significantly dysregulated of the pan-cancer pathways (Figure S2).

These data highlighted key dysregulated genes shared across different cell types in BOS [28], that are also some of the most highly dysregulated genes in the AML-*ASXL1* dataset, revealing potential biomarkers and therapeutic targets. These genes include *Vang-like 2 (VANGL2)*, a member of the planar cell polarity pathway [48], *glutamate ionotropic receptor kainate type subunit 5 (GRIK5)*, a pre- and post-synaptic receptor for glutamate, a crucial excitatory neurotransmitter of the central nervous system [28, 49], and *gremlin 2 (GREM2)*,* a* bone morphogenetic protein antagonist involved in developmental processes and tissue differentiation [50], as well as the transmembrane low-density lipoprotein receptor-related proteins 5 and 6 (*LRP5* and *LRP6*), which are key components of the Wnt signaling pathway. In particular, we identified significant upregulation of *VANGL2* (BOS log2FC = 3.80, AML-*ASXL1* log2FC = 4.24) (Fig. 2C) and *GRIK5* (BOS log2FC = 3.83, AML-*ASXL1* log2FC = 4.67) (Fig. 2D) in both BOS and AML-*ASXL1* and significant downregulation of *GREM2* in both conditions (BOS log2FC=-2.48, AML-*ASXL1* log2FC=-1.67) (Fig. 2E; Table 2). Similarly, we identified significant upregulation of *LRP5* (BOS log2FC = 1.64, AML-*ASXL1* log2FC = 2.65) (Fig. 2F) and *LRP6* (BOS log2FC = 1.63, AML-*ASXL1* log2FC = 2.21) (Fig. 2G) in both BOS and AML-*ASXL1* samples.

These DEGs were previously established in our study across different tissues in BOS as key biomarkers [28]. An independent study comparing AML-*ASXL1* to AML without *ASXL1* variants also identified *VANGL2*, *LRP5* and *LRP6* as three of the most significantly upregulated genes using a limited microarray probe-set [51], supporting that aberrant Wnt signaling occurs in the presence of *ASXL1* pathogenic variants. These expression changes suggest roles for these genes in the pathophysiology of disorders associated with *ASXL1* variants and highlight their potential as biomarkers for *ASXL1* variants. Interestingly, we identified significant upregulation of *ASXL1* expression in AML-*ASXL1* samples compared to tissue-matched controls (log2FC = 0.46, p_adj_= 1.11E-02) but no significant dysregulation of *ASXL1* expression in BOS (Fig. 2H). Overall, our study demonstrates a clear link between *ASXL1* variants driving aberrant Wnt-signaling in both BOS and AML.

### Analysis of polycomb group (PcG) target genes in BOS and AML-*ASXL1* samples

We examined the expression of Polycomb group (PcG) target genes in BOS and AML-*ASXL1* samples to determine whether these genes were differentially expressed in the context of *ASXL1* mutations (Table S3). Drawing from the list of PcG target genes identified by Bracken et al. (2006), we found that only two genes were significantly differentially expressed across both diseases: *Special AT-rich Sequence Binding Protein 1* (*SATB1*) and *Transcription Factor 7* (*TCF7*). *SATB1* was significantly upregulated in both BOS (log2FC = 0.45) and AML-*ASXL1* (log2FC = 1.07). In contrast, *TCF7* was significantly upregulated in BOS (log2FC = 0.81) but downregulated in AML-*ASXL1* (log2FC = -1.25). While SATB1 and TCF7 were the only two PcG target genes that showed significant dysregulation in BOS, a total of 18 out of the 40 target genes were significantly dysregulated in AML-*ASXL1*.

### Analysis of known protein interactors with ASXL1 in BOS and AML-ASXL1

To investigate potential direct effects of *ASXL1* mutations on gene expression, we analyzed publicly available gene sets of known ASXL1 interactors. From the 286 ASXL1 interactions identified in the Biological General Repository for Interaction Datasets (BioGRID) database [52], we identified 151 genes that were significantly dysregulated in AML-*ASXL1*, compared to only 17 in BOS. Notably, 9 of the 17 significant DEGs in BOS were also dysregulated in AML-*ASXL1*, with 8 of these genes showing upregulation in both conditions. The commonly upregulated genes included: *phosphoglycerate dehydrogenase (PHGDH)*,* androgen receptor (AR)*,* solute carrier family 25 member 15 (SLC25A15)*,* RNA binding fox-1 homolog 2 (RBFOX2)*,* gem nuclear organelle associated protein 4 (GEMIN4)*,* chaperonin containing TCP1 subunit 3 (CCT3)*,* inosine monophosphate dehydrogenase 2 (IMPDH2)*, and *phosphoribosylaminoimidazole carboxylase and phosphoribosylaminoimidazole- succinocarboxamide synthase (PAICS)*.

Additionally, Li et al. (2017) identified 182 ASXL1-interacting proteins through mass spectrometry in HEK293T cells transfected with full-length ASXL1 and C-terminal truncated ASXL1 [53]. Only 7 of these genes were significantly dysregulated, and 5 of them also showed significant dysregulation in AML-ASXL1 blood and bone marrow. Importantly, all 5 genes were dysregulated in the same direction in both diseases.

Furthermore, our analysis of the PRC2 interactome revealed that 9 out of 15 genes in this gene set were significantly dysregulated in AML-*ASXL1*, while none reached significance in the BOS samples [54, 55]. Notably, 3 of the genes identified in the PRC2 interactome set overlapped with the ASXL1 interactome set identified by BioGRID.

### DNA methylation driven de-repression of *HOX* genes identified across BOS and AML-*ASXL1* samples

Our integrated analyses of DNAm leveraged our previously published BOS-specific DNAm episignature [33] to distinguish pathogenic *ASXL1* variants from normotypic matched controls and variants of uncertain significance (VUS) in *ASXL1* [33]. To assess whether AML-*ASXL1* samples and BOS samples shared DNAm signatures and epigenetic changes, we obtained Illumina 450K DNAm data for AML samples (*n* = 6) from TCGA on the GDC repository [30, 56]. This comprised individuals harboring somatic variants in *ASXL1* (*n* = 3, AML-*ASXL1*) or somatic variants in other genes (*n* = 3, AML).

**Fig. 3:**
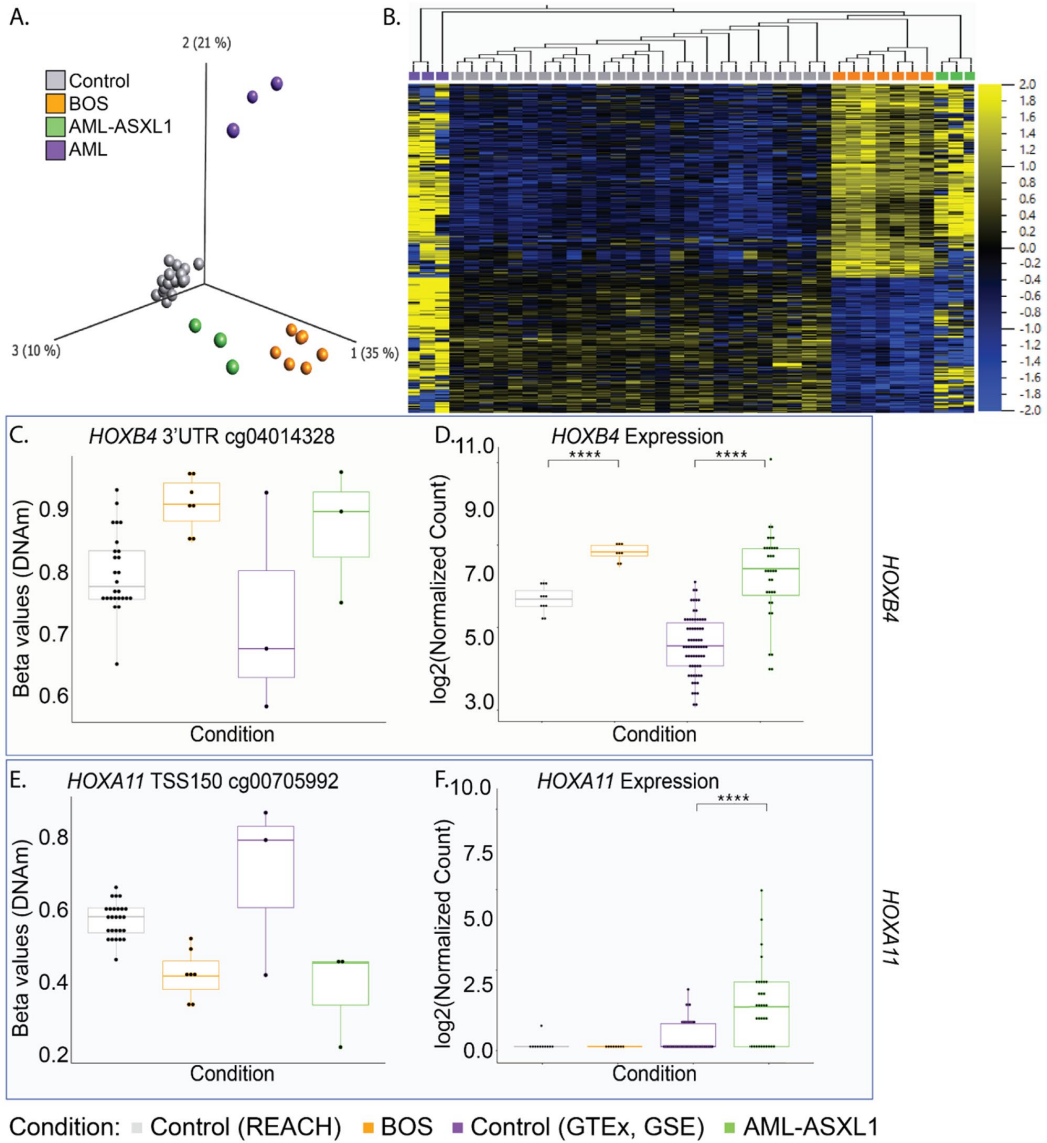
Epigenetic landscape of *ASXL1* variants in BOS and AML-*ASXL1* highlights de-repression of *HOX* genes. (**A**) Principal component analysis (PCA) plot demonstrates the closer clustering of acute myeloid leukemia with *ASXL1* variants (AML-*ASXL1*) samples (green, *n* = 3) with Bohring-Opitz syndrome (BOS) samples (orange, *n* = 8) compared to AML samples with somatic variants in other genes (AML, purple, *n* = 3), and control samples (gray, *n* = 26), illustrating shared epigenetic landscapes driven by *ASXL1* variant status. (**B**) Heatmap representing DNA methylation patterns (DNAm) using the 413 episites identified in the BOS DNAm episignature depicts a consistent clustering pattern of the unique clustering of AML-*ASXL1* samples alongside BOS samples. (**C**) DNAm β values for HOXB4 3’UTR at CpG site cg04014328 highlight the hypermethylation in both BOS and AML-*ASXL1* patients compared to controls and other AML samples. (**D**) RNA-sequencing (RNA-seq) data demonstrates significant upregulation of *HOXB4* in BOS and AML-*ASXL1* compared to their respective controls. (**E**) DNAm β values for HOXA11 TSS1500 at CpG site cg00705992 highlight the hypomethylation in both BOS and AML-*ASXL1* patients compared to controls and other AML samples. (**F**) RNA-seq data demonstrates significant upregulation of *HOXA11* in AML-*ASXL1* but not in BOS

We compared DNAm episignature profiles of blood samples from healthy controls (*n* = 26), BOS (*n* = 8), AML-*ASXL1* (*n* = 3), and AML controls (*n* = 3). PCA based on 413 CpG sites of the BOS DNAm episignature [33] revealed significant differences between the leukemia subtypes. AML-*ASXL1* samples clustered distinctly from other AML samples without *ASXL1* variants and, instead, clustered more closely with BOS samples (Fig. 3A). Among the 413 BOS episignature CpGs, 90 CpG sites corresponded to transcriptional start sites (TSS), including regions 200 bp upstream (TSS200) or 1500 bp upstream (TSS1500) (Table S4). Unsupervised clustering of CpG methylation across the 413 BOS episignature CpG sites also showed that AML-*ASXL1* clusters alongside BOS samples and remains distinct from AML controls (Fig. 3B). This suggests that *ASXL1*-driven epigenetic alterations transcend disease type in defining DNAm patterns.

We examined the methylation status of posterior homeobox A (*HOXA*) genes, given that the expression of these genes are known to be regulated by *ASXL1* [25, 57–59], and *HOXB3* and *HOXB4* [60] (Table 4). The latter two genes are expressed in hematopoietic stem cells (HSCs) and progenitors as “master genes in early hematopoiesis”, and exhibit lineage and differentiation stage-restricted expression [61–63].

**Table 4:**
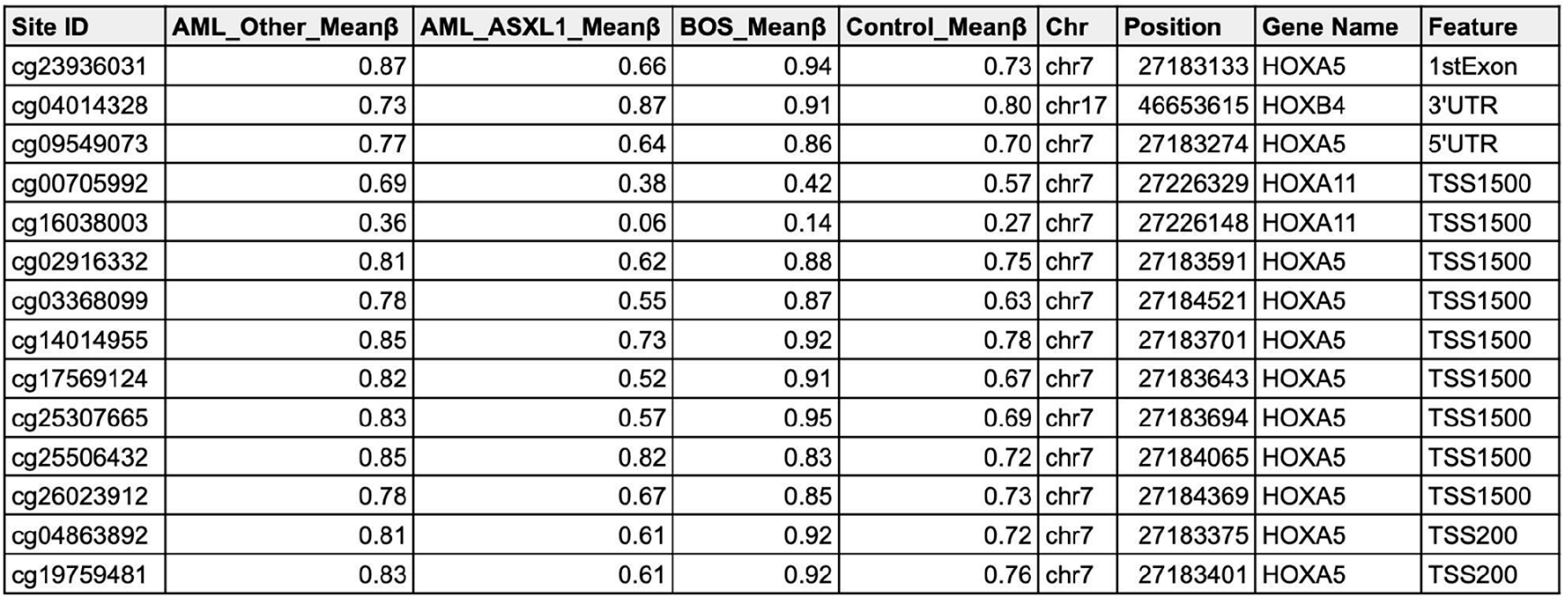
DNA methylation analysis at HOX gene sites across different sample groups

Average mean DNA methylation (DNAm) beta (β) values at HOX gene sites for four sample groups: AML with non-*ASXL1* variants (AML-other, n=3), AML with *ASXL1* variants (AML-*ASXL1*, n=3), Bohring-Opitz syndrome (BOS, n=8), and controls (n=26).

The *HOXB4* 3’UTR was hypermethylated in BOS and AML-*ASXL1* samples compared to respective controls (Fig. 3C). Consistent with findings that 3’UTR methylation correlates positively with gene expression [64], RNA-seq data showed significant upregulation of *HOXB4* (BOS log2FC = 1.41, AML-*ASXL1* log2FC = 1.89) (Fig. 3D). Significant upregulation was also observed in *HOXB3* (BOS log2FC = 1.34, AML-*ASXL1* log2FC = 2.48) (Figure S3A). While overexpression of *HOXB4* has been shown to drive enhanced HSC regeneration, deficiency of *HOXB3* or *HOXB4* leads to defects in proliferative ability of hematopoietic progenitors [65]. Furthermore, one of the key targets of *HOXB4* is Wnt signaling [66]. Our investigation of Wnt signaling revealed significant upregulation of Wnt signaling coreceptors *LRP5* and *LRP6* in both BOS and AML-*ASXL1* samples (Fig. 2F and G).

DNAm analysis further identified hypomethylation at specific CpG sites upstream of the *HOXA11* TSS (cg00705992 and cg16038003) in BOS and AML-*ASXL1* samples, supporting literature that truncating *ASXL1* variants lead to derepression of posterior *HOXA* genes (Fig. 3E and S4A). Interestingly, while *ASXL1* variants drove significant upregulation of *HOXA11* transcript expression (log2FC = 3.87, p_adj_=1.33E-06) in AML-*ASXL1*, there was no significant dysregulation of *HOXA11* in BOS. Similar trends were identified in other posterior *HOXA* genes including *HOXA5* (Figure S3B and S4B) and *HOXA9* (Figure S3C); there was no significant dysregulation in BOS samples.

These findings underscore the pervasive influence of *ASXL1* variants in modulating the epigenetic and transcriptomic landscapes across diseases, promoting abnormal gene expression and signaling pathways crucial for disease pathology.

### Transcriptomic and epigenomic differences are not driven by differences in cell-type proportion

To ensure that our epigenetic and transcriptomic findings were not influenced by differential blood cell type distributions, we performed a detailed analysis of cell type proportions in the blood samples from BOS and control individuals. This analysis was essential given the heterogeneous nature of whole blood samples, which comprises multiple immune cell types. In our DNAm (Figure S5A) and RNA-seq data (Figure S5B), we compared the proportions of several key immune cell types between BOS and control samples. Table S4 provides a summary of the CIBERSORTx cell type deconvolution results applied to the RNA-seq data. B cells (p_adj_=1.00), NK cells (p_adj_=1.00), monocytes (p_adj_=1.00), and neutrophils (p_adj_=0.678) showed no significant differences, while T cells had a slight significant difference (p_adj_=4.34E-02). Specifically, this was driven by a significant increase in CD4 + T cells (p_adj_=1.97E-04), which play critical roles in effective anti-tumor immunity [67], and not CD8 + T cells (p_adj_=0.44). However, this difference was not identified in the DNAm data. These findings suggest that the epigenetic and transcriptomic findings are not driven by variations in blood cell type proportions.

### Differential RUNX3 isoform expression in BOS and AML-*ASXL1*

**Fig. 4:**
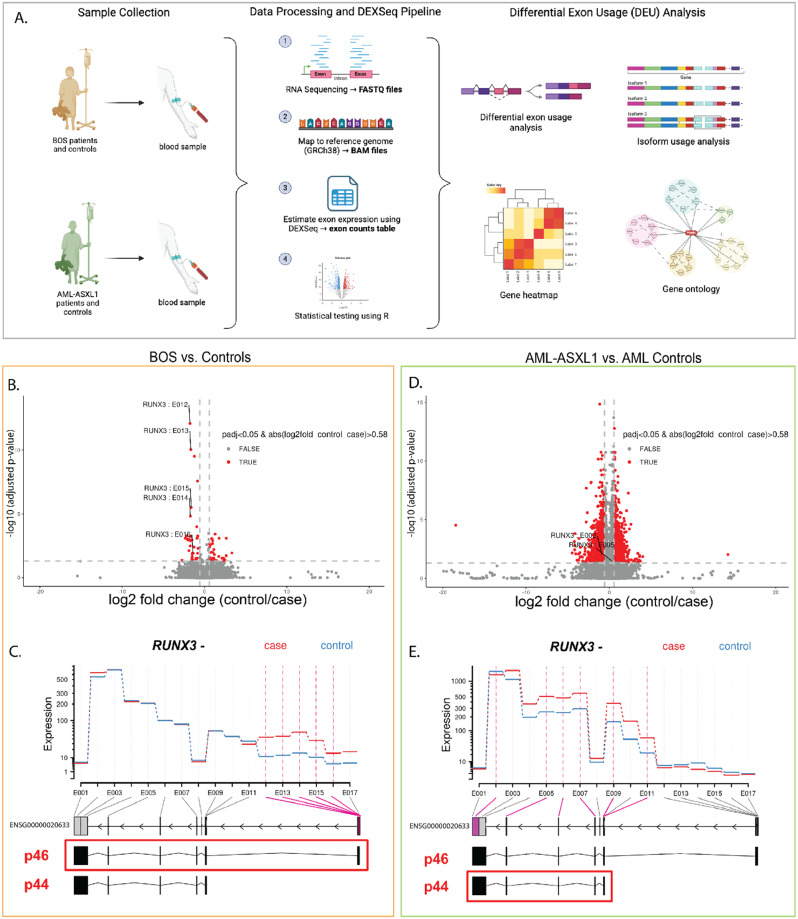
Differential exon usage (DEU) analysis of BOS and AML-*ASXL1* blood reveals differential isoform usage of *RUNX3* between diseases. (**A**) Using DEXSeq, we obtained exon counts and performed DEU analysis in *ASXL1*-variant disease samples compared to controls, isoform usage and gene ontology analysis. An integrated analysis was conducted between BOS and AML-*ASXL1* samples. We performed DEU analysis for BOS compared to controls (orange outline). (**B**) Fold change plot of significant DEUs (p_adj_ < 0.05) in BOS patients compared to controls highlights *RUNX3* as a key affected gene with multiple DEUs. (**C**) Fitted expression exon usage plot for RUNX3 in BOS blood samples, with significant DEUs indicated with a pink line. Cases are shown in red and controls in blue. The boxed transcript highlights the primary transcript, the longer p46 transcript, observed in BOS blood. We also performed DEU analysis for AML-*ASXL1* (green outline). (**D**) Fold change plot of significant DEUs (p_adj_ < 0.05) in AML-*ASXL1* patients compared to controls also highlights *RUNX3* as an affected gene with multiple DEUs. (**E**) Fitted expression exon usage plot for *RUNX3* in AML-*ASXL1* blood. Cases are shown in red and controls in blue. The boxed transcript highlights the primary transcript, the shorter p44 transcript that does not have the first exon, observed in AML-*ASXL1*

In developmental processes, one of the key regulatory mechanisms is mediated through RNA splicing. Differential isoform usage is thought to be a key mechanism driving cell-specific differentiation and disease [68–70]. To assess potential isoforms, we performed differential exon usage (DEU) analysis in BOS blood (*n* = 8), blood controls (*n* = 10), AML-*ASXL1* blood (*n* = 4), and AML blood controls (*n* = 6). Using our mapped RNA-seq data, we estimated exon expression using DEXSeq [39] and performed statistical testing to determine DEU in BOS and AML-*ASXL1* (Fig. 4A).

Exon counts were calculated for a total of 597,773 exonic regions in 56,940 genes and analyzed for DEU (Figure S6). A total of 88 significant exonic regions from 63 affected genes were identified in BOS blood (p_adj_ < 0.05). Some of the most significant DEU from BOS blood were in the following genes: *runt-related transcription factor 3 (RUNX3)*,* plasmolipin (PLLP)*,* ST6 beta-galactoside alpha-2*,*6-sialyltransferase 1 (ST6GAL1)*,* serpin family F member 1 (SERPINF1)*, and *pleckstrin homology* and *RhoGEF domain containing G6 (PLEKHG6*) (Table S6). Many of these genes were also identified as DEGs in the multi-omics study by Lin et al. (2023) and are involved in embryonic or brain development and neuronal differentiation [28, 71, 72]. In AML-*ASXL1* blood, a total of 11,624 significant DEU bins from 4521 affected genes were identified (padj < 0.05). Many of the exons with significant DEU in AML-*ASXL1* blood had immune- or cancer-related function, such as *TBL1X/Y related 1 (TBL1XR1)*,* DEAD-box helicase 42 (DDX42)*,* Rho associated coiled-coil containing protein kinase 1 (ROCK1)*,* neurobeachin like 2 (NBEAL2)*, and *UDP-glucose pyrophosphorylase 2 (UGP2)* (Table S7). Across these two analyses, 15 affected genes were shared, most notably *RUNX3*.

We next asked whether *ASXL1* displayed DEU in BOS or AML-*ASXL1*. We did not observe significant DEU within *ASXL1* itself for BOS blood or fibroblast (Figure S8A-B). Unsupervised clustering of *ASXL1* exon usage in BOS blood and fibroblast compared to controls showed grouping by tissue type, indicating that tissue specificity, not disease status, drives exon usage for *ASXL1* in BOS (Figure S8D). In AML-*ASXL1* blood, while we observed DEU for multiple exons bins of *ASXL1*, these did not correlate with known *ASXL1* isoforms (Figure S8C).

While there are shared cellular pathways, these two disorders have distinct isoform expression, even when comparing the same tissue type. Notably, our DEU analysis identified *RUNX3* among the most significant DEUs between *ASXL1* mutant and control samples in both BOS blood (Fig. 4B) and AML-*ASXL1* blood samples (Fig. 4D). While there were several significant DEUs in both BOS blood and AML-*ASXL1* blood, these DEUs did not overlap across disorders. Interestingly, the 5 exons bins that were significantly upregulated in BOS samples corresponded to higher usage of the first exon, exon 1 of *RUNX3* (Fig. 4B-C, Table S8). On the contrary, we observed DEU of 6 exon bins in *RUNX3* for AML-*ASXL1* blood samples (Fig. 4D, Table S7) which corresponded to higher usage of the last exons (Fig. 4D, Table S8), and *lower* usage of the first exon (Fig. 4E). Therefore, opposite effects on exon usage were observed in BOS and AML-ASXL1 blood, which highlights that there do exist clear isoform differences between these two disease states. Finally, DEU of *RUNX3* was also identified in BOS fibroblasts samples (Figure S7A) [28] and unsupervised clustering revealed that this DEU was correlated with disease status over tissue type.

*RUNX3* is known to have two main transcripts expressed in blood cells— p46 which is expressed from the distal P1 promoter and includes the first exon of the gene, and p44 which is expressed from the proximal P2 promoter and does not include the first exon [73]. The distal P1 promoter has previously been shown to have a role in CD8 + T-cell function; on the other hand, the proximal P2 promoter is often hypermethylated and epigenetically inactivated in solid tumors, leading to inefficient expression compared to the P1 promoter [74, 73].

Our results suggest that the p44 transcript is more highly expressed in AML-*ASXL1* blood, while the p46 transcript is more highly expressed in BOS blood.

## Discussion

In this study, we aimed to address a fundamental question: do *ASXL1* variants exert common molecular effects across distinct disease types? We examined the epigenetic and transcriptomic landscapes associated with *ASXL1* variants in BOS and AML, diseases with different clinical manifestations - BOS as a congenital disorder characterized by developmental delays and multiple malformations, and AML as a bone marrow malignancy - and identified several shared features. Both diseases exhibited aberrant activation of Wnt signaling and disrupted posterior *HOX* gene expression. Notably, we observed differences in *RUNX3* isoform usage; the longer isoform, p46, may act as a tumor suppressor in BOS [75, 76] while a shorter *RUNX3* isoform predominates in AML. Targeting the longer isoform of RUNX3 may hold therapeutic potential to mitigate the malignant potential of HSCs. This study highlights the shared molecular disruption driven by high-effect *ASXL1* variants and suggests potential therapeutic pathways, offering a rationale for the development of targeted therapies applicable to *ASXL1* related diseases.

Epigenetic variations are thought to be a major driver of differentiation and maintenance of cell-specification [77] in these genes could drive divergent effects across cell types and diseases. While traditional approaches often focus on directly correcting or targeting the mutated gene, our data, along with previous studies [28, 33], show that the same genetic variant can converge on common pathways across different diseases and cell types [28]. We propose that targeting these shared pathways presents an alternative approach towards treatment for multiple disorders caused by *ASXL1* variants. Understanding the interplay of genetic variants, cell-type, genetic background, and disease state can help identify improved therapeutic biomarkers and precision targeted therapies that supersede clinical disease features. For patients with disorders that affect multiple organ systems, such as BOS, the high barriers to effective gene therapy require parallel approaches that target the shared pathways underlying these conditions. This strategy could lead to more versatile and broadly applicable treatments, and better management of diseases associated with *ASXL1* variants.

### Differential isoform usage associated with *ASXL1* mutations

Our study on DEU serves as a proxy for true isoform presence, and indicates that disease states can be heavily influenced by isoform usage. While elements of *ASXL1*-driven epigenetic and transcriptomic dysregulation are shared among these disorders, RUNX3 isoform usage appears to be distinct and closely tied to disease pathology. *ASXL1* mutations have been shown to induce alternative splicing in mutated cell lines [78], however, determining whether the alternative splicing events of *RUNX3* in BOS and AML-*ASXL1* are primary effects of *ASXL1* mutations is beyond the scope of this study.

One possible hypothesis is that *ASXL1* mutations may be accompanied by additional mutations in splicing-related genes that directly control *RUNX3* isoform usage. Alternatively, *ASXL1* mutations may indirectly affect pathways that result in alternate isoform usage. For example, additional spliceosomal mutations and age-related changes in the expression of RNA-binding proteins and RNA modifications could explain the observed differences in RUNX3 isoform usage between BOS and AML-*ASXL1*.

### Direct transcriptomic effects of *ASXL1* mutations across BOS and AML-*ASXL1*

In our study, we examined the relationship between *ASXL1* mutations and the expression of a set of 40 PcG target genes previously identified by Bracken et al. (2006) [79]. The limited number of significant dysregulated genes in BOS (only 2 out of 40) compared to AML-*ASXL1* (18 out of 40) may reflect the inherent limitations of a smaller sample size associated with BOS, a rare disease.

One of the polycomb complexes that *ASXL1* is known to interact with is PRC2, which plays a critical role in gene regulation through epigenetic mechanisms. Mutations in *ASXL1* can disrupt the normal function of PRC2, and loss of function mutations have been shown to cause decreased H3K27me3 levels at target genes. Our analysis of the PRC2 interactome gene set published in previous studies [54, 55] revealed that 9 out of 15 genes showed significant dysregulation in AML-*ASXL1* compared to none in BOS. This discrepancy suggests a more pronounced effect of *ASXL1* mutations on gene regulation within the context of leukemia.

Notably, *SATB1* and *TCF7* are significantly dysregulated in both diseases, and may be a result of direct interactions with ASXL1. The consistent upregulation of *SATB1* in both BOS and AML-*ASXL1* suggests its role as a key regulator of chromatin architecture and gene expression relevant to both conditions. SATB1 is a chromatin organizer and transcription factor which is enriched at gene promoters and enhancers involved in long-range chromatin interactions [80–82].

In contrast, the significant upregulation of *TCF7* in BOS and downregulation in AML-*ASXL1*, suggests context-dependent regulatory functions. *SATB1* plays a crucial role in maintaining appropriate transcriptional programs within naive CD8 + T cells, and *TCF7* is one of the key naive transcription factors targeted by *SATB1* [80]. Intriguingly, other key naive transcription factors regulated by SATB1 binding, including *BCL6*,* BCL11B*,* FOXO1*, and *LEF1*, also exhibited significant downregulation in AML-*ASXL1* while being upregulated in BOS. This pattern may reflect distinct cellular environments and the varying influences of *ASXL1* mutations in different disease states.

In this study, we also examined dysregulation of gene expression in gene subsets known to interact with ASXL1. The limited number of dysregulated genes in BOS may be a consequence of smaller sample size. Interestingly, 5 out of 7 ASXL1-interacting proteins dysregulated in BOS blood were also differentially expressed in AML-*ASXL1*, and dysregulated in the same direction, supporting the possibility of shared pathogenic mechanisms, despite the different clinical presentations.

### Limitations of this study

There remain some key limitations to our study. First and foremost, AML is a heterogeneous disorder with multiple genetic variants and aberrations present in every sample. We suspect that the heterogeneity, even in the presence of the *ASXL1* variant, requires a larger sample size to detect true and consistent effects due to *ASXL1* variants. However, we do believe that patient-derived germline variants as seen in BOS provide a clean background for isolation of the genetic effect of *ASXL1* variants and prioritization of putative targets that are common to both diseases. Moreover, the presence of epigenetic and transcriptional changes observed in both BOS and AML-*ASXL1* samples serve to highlight the strong and consistent effect of *ASXL1* on gene expression, which supersedes even the effects of tissue [28] and disease [51, 83].

### Research and clinical implications of this study

RASopathies encompass a range of genetic syndromes such as Noonan syndrome, Costello syndrome, and neurofibromatosis type 1, and are characterized by variants in multiple genes that regulate the activity of the RAS/MAPK signaling pathway [84]. These variants lead to hyperactive signaling, resulting in developmental abnormalities, cardiovascular defects, and an increased risk of certain cancers [84]. The successful grouping of these disorders has allowed for targeting of shared pathways and drug repurposing which not only leverages existing drugs with known safety profiles such as lovastatin and everolimus but also accelerates the development of targeted therapies, reducing the time and cost associated with bringing new treatments [85–90]. Mouse studies and early clinical trials have suggested that targeted inhibition of the RAS/MAPK pathway can mitigate some of the severe manifestations of these RASopathies [90–94]. We believe that a common approach can be used to ameliorate some of the clinical and molecular effects of chromatinopathies.

One intriguing extension from our analysis is the potential to repurpose or harness novel Wnt inhibitors or chromatin modifying drugs to ameliorate the effects of *ASXL1* variants in a range of diseases, including BOS and subtypes of AML. Our analysis highlights the strong influence of *ASXL1* variants on transcriptional regulation, with over 500 genes differentially expressed in both AML-ASXL1 and BOS data sets, indicating a shared molecular dysfunction. This shared dysregulation is particularly evident in genes involved in epigenetic regulation, chromatin modification, and the canonical Wnt signaling pathway, which are critical for cell fate determination and proliferation. Our findings suggest a common thread in the molecular mechanisms of *ASXL1* variants– through dysregulation of Wnt signaling pathways and posterior *HOX* gene expression.

### Modulating Wnt-signaling in *ASXL1*-mutated diseases

Our data highlighted the aberrant activation of Wnt signaling associated with *ASXL1* variants across disease types. Our transcriptomic integration showed that the Wnt signaling co-receptors *LRP5* and *LRP6* (Fig. 2A, F and G) and non-canonical Wnt signaling through *VANGL2* are all upregulated at the transcriptomic level and, in BOS samples, also at the protein level [28]. VANGL2 is a key transmembrane protein in the planar cell polarity pathway and is thought to drive cellular orientation in 3D space and migration patterns, both of which are pivotal in both oncogenic transformations and developmental anomalies. Our work provides an orthogonal validation of previous RNA-microarray data in AML-*ASXL1* that also identified upregulation of *LRP6* and *VANGL2* [51] using a less sensitive approach. This independent data and analysis highlights that despite the different genomic technologies, disease states, genetic background of the cells and differences in samples, *ASXL1* variants drive a shared effect among these and other genes. To our knowledge, there are three human and disease-specific data sets in which transcriptomic, and in the case of BOS, protein data, confirm the abnormal activation of Wnt signaling markers associated with *ASXL1* variant [28, 33, 95]. Overall, the consistent dysregulation of key genes across different diseases suggests that these findings are not merely artifacts of disease-specific processes but are potentially pivotal drivers of pathophysiology associated with *ASXL1* variants. The discovery of these cross-disease biomarkers offers a promising avenue for further research and development of diagnostic tools and therapeutic strategies. Therapeutics targeting *VANGL2* and *LRP6* could potentially be used as targets for ASXL1-precision therapies.

The Wnt signaling pathway has been a tantalizing target for drug development in a variety of solid tumors and leukemias, but to date there are no FDA approved drugs that are targeted towards tumors with over-active Wnt-signaling. One challenge is that these treatments often engender significant side effects associated with modulation of this central pathway [96]. Currently, there are multiple phase I and II clinical trials that target different aspects of Wnt pathways such as the beta-catenin destruction complex [96, 97] or direct blockage of Beta-catenin with its binding partners. To date, there are no approved precision therapies for patients with *ASXL1* variants, and careful modulation of the canonical Wnt signaling pathway represents a potential therapeutic option for BOS patients and for AML-*ASXL1.* Studies in preclinical models, such as mice and rats, are needed to understand the interplay between these Wnt-signaling pathways and *ASXL1* variants and potential off target effects.

### Decreased malignant transformation in BOS hint at potential biomarkers for AML-*ASXL1*

*ASXL1* variants in AML lead to dysregulation of genes involved in patterning in hematopoiesis and myeloid differentiation such as *HOXA* genes that were both differentially expressed and methylated in AML-*ASXL1*. These data are consistent with previous work showing that *ASXL1* variants disrupt the normal repression of posterior *HOXA* genes during myeloid cell differentiation [25, 33, 98]. This dysregulation likely drives the proliferation of immature myeloid cells, a hallmark of AML. Conversely, in BOS, while similar DNAm changes were identified, the corresponding transcriptional dysregulation was not observed, suggesting that other additional factors are required for transformation. Clinically, no BOS patients have been diagnosed with AML, but they do have an increased risk of Wilms tumor, a pediatric kidney tumor [99].

The absence of myeloid dysplasia in BOS, despite the presence of *ASXL1* variants, suggests that additional factors are necessary to trigger leukemogenesis. These factors could include secondary genetic variants, epigenetic changes, or specific microenvironmental or tissue-specific cues that are absent in BOS patients but present in the context of AML. In AML, where *ASXL1* variants are only in the leukemia stem cells (LSCs), there is a potential imbalance in paracrine signaling factors and receptors between the microenvironment (no *ASXL1* variant) and the LSC (with *ASXL1* variant).

While we observe many similarities in the transcriptional profiles between these two diseases, the genes that are dysregulated in opposite directions might provide therapeutic targets and biomarkers– centered around turning AML-*ASXL1* profile closer to that seen in BOS blood, thereby ameliorating the malignant potential. We found that BOS cells primarily expressed the longer p46 isoform of RUNX3, which plays a tumor suppressor role and might explain the decreased transformation in BOS compared with AML-*ASXL1* that expresses the shorter p44 isoform. Reactivation of the longer *RUNX3* isoform in AML might provide a potential therapeutic strategy in AML-*ASXL1*.

Mouse models of *ASXL1* mutations show defects in HSC proliferation and myeloid differentiation [100, 101] in addition to disrupted development. While these models provide valuable insight, they do not always fully reflect the complexities of human diseases. Notably, mouse models typically feature homozygous deletion mutants, while human variants are heterozygous and truncating variants. Additionally, while there are some similarities in hematopoiesis between humans and mice, there are limited effective mouse models for myeloid leukemogenesis, emphasizing significant differences in disease manifestation. Importantly, ASXL1 mutations in mouse models do not lead to myeloid transformation without the presence of additional mutations [102]. This distinction is particularly evident when considering BOS patients, who are generally much younger compared with the average AML patient. The absence of leukemic transformation in BOS patients is intriguing and may suggest novel therapeutic approaches in AML-*ASXL1*.

## Conclusion

This comparative analysis provides a deeper understanding of the complex molecular underpinnings of *ASXL1* variants in BOS and AML, highlighting shared and unique molecular features. By delineating the genetic, epigenetic, and transcriptomic impacts of these variants, our study not only advances the understanding of the molecular pathology of these conditions but also sets the stage for the development of targeted therapeutic strategies that address the specific molecular alterations associated with *ASXL1* variants. Novel or repurposed therapies targeted against the effects of *ASXL1* can be used regardless of clinical presentation: germline or somatic, and provides a pathway to drug development even for the rarest conditions. Overall, our study advocates for a gene-centric approach in understanding the molecular basis of diseases associated with *ASXL1* variants.

Our study contributes to a broader understanding of how genetic variants can influence disease across traditional phenotypic boundaries. This not only challenges existing paradigms of disease classification but also opens new avenues for innovative therapeutic strategies that are driven by molecular commonalities rather than solely clinical features. This gene-centric perspective could redefine therapeutic strategies and promote the development of more precise and effective treatments for genetically driven disorders.

## Electronic supplementary material

Below is the link to the electronic supplementary material.

Supplementary Material 1

## Data Availability

The datasets analyzed during the current study were collected from our previous study and are available in the GEO repository, under accession number GSE230685 and GSE230696. A subset of the DNA methylation (DNAm) datasets generated during the current study are not publicly available due to institutional ethical restrictions but are available from the corresponding author on reasonable request to authors.Illumina 450 K DNAm data and RNA-sequencing (RNA-seq) data for AML and AML-ASXL1 blood samples were acquired from The Cancer Genome Atlas (TCGA) program, available on the Genomic Data Commons (GDC) repository, accessible at https://portal.gdc.cancer.gov/projects/TCGA-LAML. Transcriptomic data for AML-ASXL1 bone marrow samples were acquired from the Beat AML cohort, available from dbGaP phs001657.v3.p1. Transcriptomic data for blood controls were obtained from the Genotype-Tissue Expression (GTEx) Portal, accessible at https://www.gtexportal.org/home/downloads/adult-gtex/bulk_tissue_expression. Transcriptomic data for bone marrow controls were obtained from the publicly available dataset by Oetjen et al., 2018, and are available in the GEO repository, under accession number GSE120444.

## Abbreviations

BOS: Bohring-Opitz syndrome
AML: Acute Myeloid Leukemia
AML-*ASXL1*: Acute Myeloid Leukemia with ASXL1 mutation
CMML: Chronic Myelomyelocytic Leukemia
MDS: Myeloid Dysplastic Syndrome
DNAm: DNA Methylation
RNA-seq: RNA-Sequencing
DEG: Differentially Expressed Genes
PRC: Polycomb Repressive Complex
PR-DUB: Polycomb Repressive Deubiquitinase
LSC: Leukemia Stem Cells
DEU: Differential Exon Usage
