# Supplementary material for: ASXL1 truncating variants in BOS and myeloid leukemia drive shared disruption of Wnt-signaling pathways but have differential isoform usage of RUNX3
Source: BMC Med Genomics. 2024 Nov 29;17:282. doi: 10.1186/s12920-024-02039-7 (PMC11606099; doi:10.1186/s12920-024-02039-7)
Supplement: Supplementary file 1 — Supplementary Material 1 [file 12920_2024_2039_MOESM1_ESM.docx]

**SUPPLEMENTAL FIGURES AND TABLES**

***ASXL1* truncating mutations in BOS and myeloid leukemia drive shared disruption of Wnt-signaling pathways but have differential isoform usage of *RUNX3***

Isabella Lin^1,2,3*^, Zain Awamleh^4*^, Mili Sinvhal^1,2,3^, Andrew Wan^1,2,3^, Leroy Bondhus^1,2,3^, Angela Wei^1,2,3,5^, Bianca E. Russell^3,6^, Rosanna Weksberg^4,7,8^, Valerie A. Arboleda^1,2,3,5,9,10^

*These authors contributed equally

^1^ Department of Pathology and Laboratory Medicine, David Geffen School of Medicine, UCLA, Los Angeles, CA, USA

^2^ Department of Computational Medicine, David Geffen School of Medicine, UCLA, Los Angeles, CA, USA

^3^ Department of Human Genetics, David Geffen School of Medicine, UCLA, Los Angeles, CA, USA

^4^ Department of Genetics and Genome Biology, The Hospital for Sick Children, Toronto, ON, Canada

^5^ Interdepartmental Bioinformatics Program, UCLA, Los Angeles, CA, USA

^6^ Department of Human Genetics, Division of Clinical Genetics, UCLA, Los Angeles, CA, USA

^7^ Department of Pediatrics, Division of Clinical & Metabolic Genetics, The Hospital for Sick Children, Toronto, ON, Canada

^8^ Institute of Medical Sciences and Department of Molecular Genetics, University of Toronto, Toronto, ON, Canada

^9^ Molecular Biology institute, UCLA, Los Angeles, CA, USA

^10^ Jonsson Comprehensive Cancer Center, UCLA, Los Angeles, CA, USA


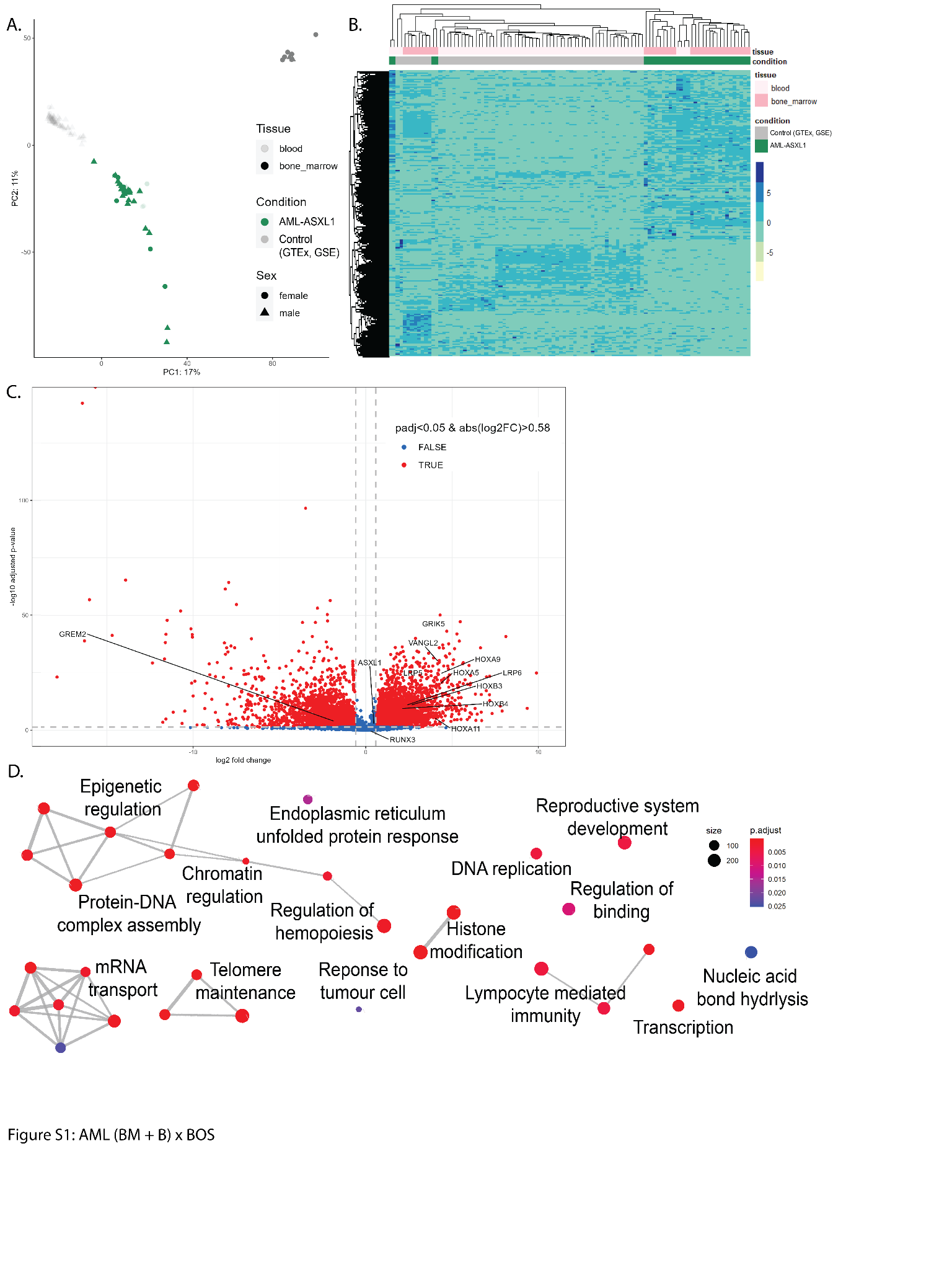


#### **Figure S1: Integrative analysis of AML-ASXL1 samples compared to controls reveals distinct transcriptomic signatures.**

**(A)** Principal component analysis (PCA) plot of all AML-ASXL1 samples sourced from BEAT-AML and TCGA, and all control samples sourced from GTEx and GSE120444. Samples are colored by condition (AML-ASXL1 = green, control = grey), shaded by tissue type (bone marrow = dark, blood = light), and the shapes represent sex (female = circle, male = triangle). The PCA plot demonstrates the distinct clustering patterns between AML-*ASXL1* and control samples. **(B)** Heatmap showing differentially expressed genes (DEGs) between AML-*ASXL1* and control samples. The heatmap highlights significant changes in gene expression patterns, providing insights into the molecular alterations associated with AML-ASXL1. **(C)** Volcano plot comparing AML-*ASXL1* samples to controls, with log_2_ fold change (log_2_FC) on the x-axis and -log10 adjusted p-value on the y-axis. Relevant genes are labeled to highlight those with significant expression changes. **(D)** Gene ontology (GO) analysis of the DEGs, illustrating the enrichment of biological processes and pathways affected in AML-*ASXL1*. This analysis identifies key functional categories that are dysregulated in AML-*ASXL1* compared to controls.

####


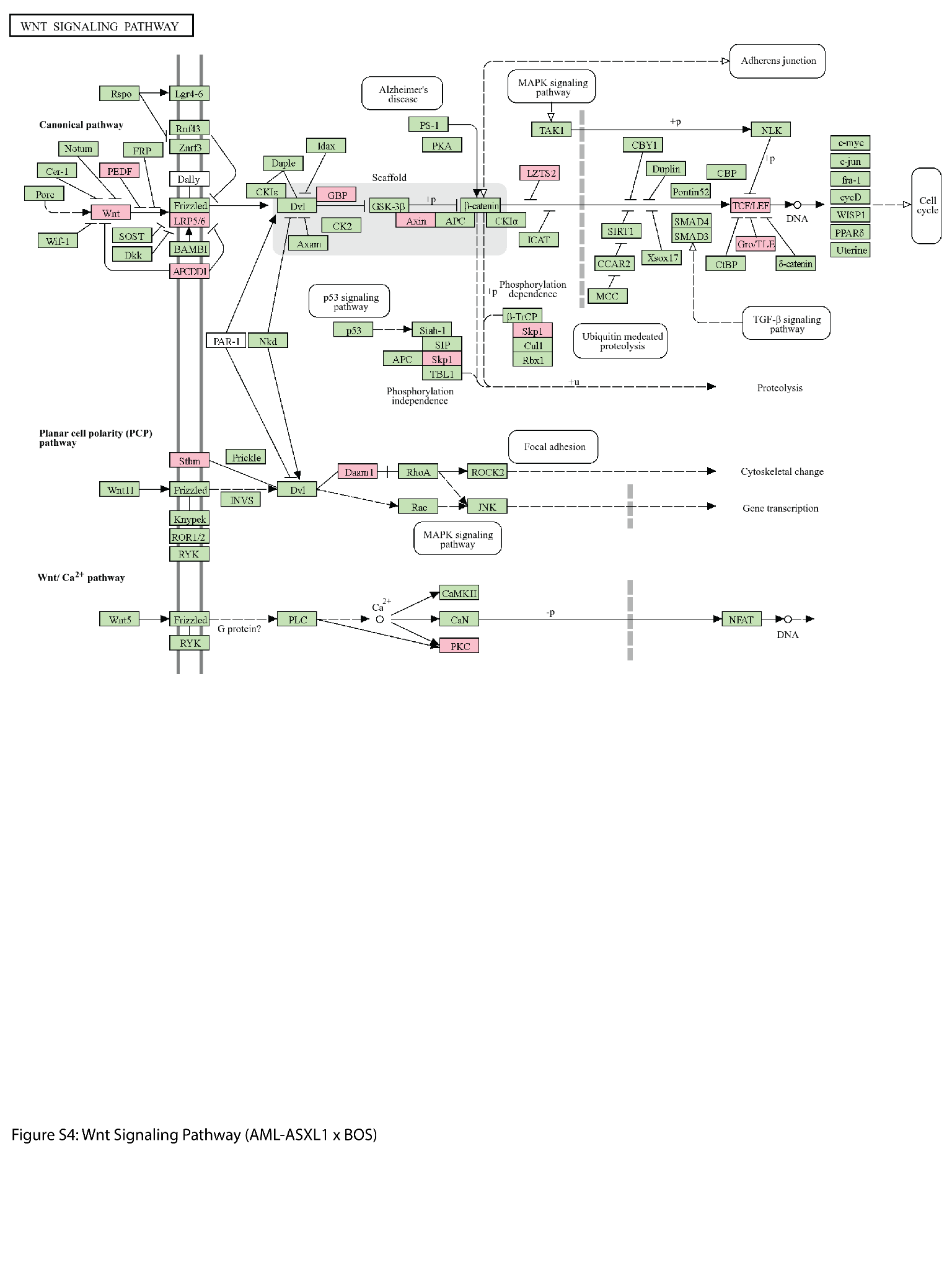


#### **Figure S2: KEGG pathway analysis highlights Wnt signaling dysregulation in AML-ASXL1 and BOS.**

*KEGG Mapper* was used to visualize differentially expressed genes (DEGs) within the canonical and non-canonical Wnt signaling pathways, identifying transcriptomic dysregulation in both AML-*ASXL1* and Bohring-Opitz syndrome (BOS). Genes highlighted in pink were significant DEGs in both AML-*ASXL1* and BOS transcriptomic analysis, and indicate multiple hits within the canonical Wnt signaling pathway.


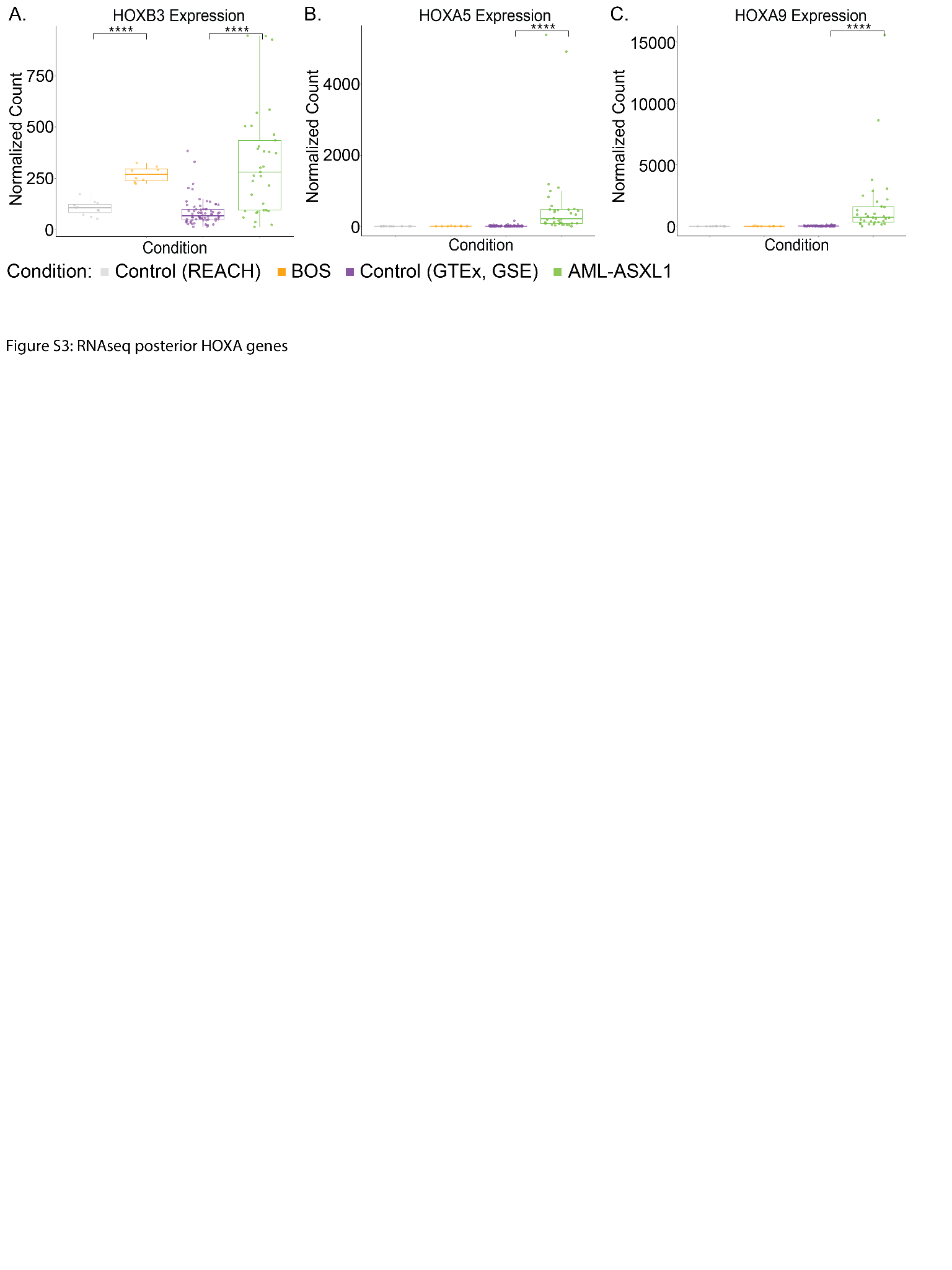


#### **Figure S3: RNAseq of HOX gene expression boxplots**

RNAseq data in BOS and AML-ASXL1 compared to their respective controls demonstrates **(A)** significant upregulation of *HOXB3* in both diseases, **(B)** significant upregulation of *HOXA5* in AML-ASXL1 only, and **(C)** significant upregulation of *HOXA9* in AML-ASXL1 only.


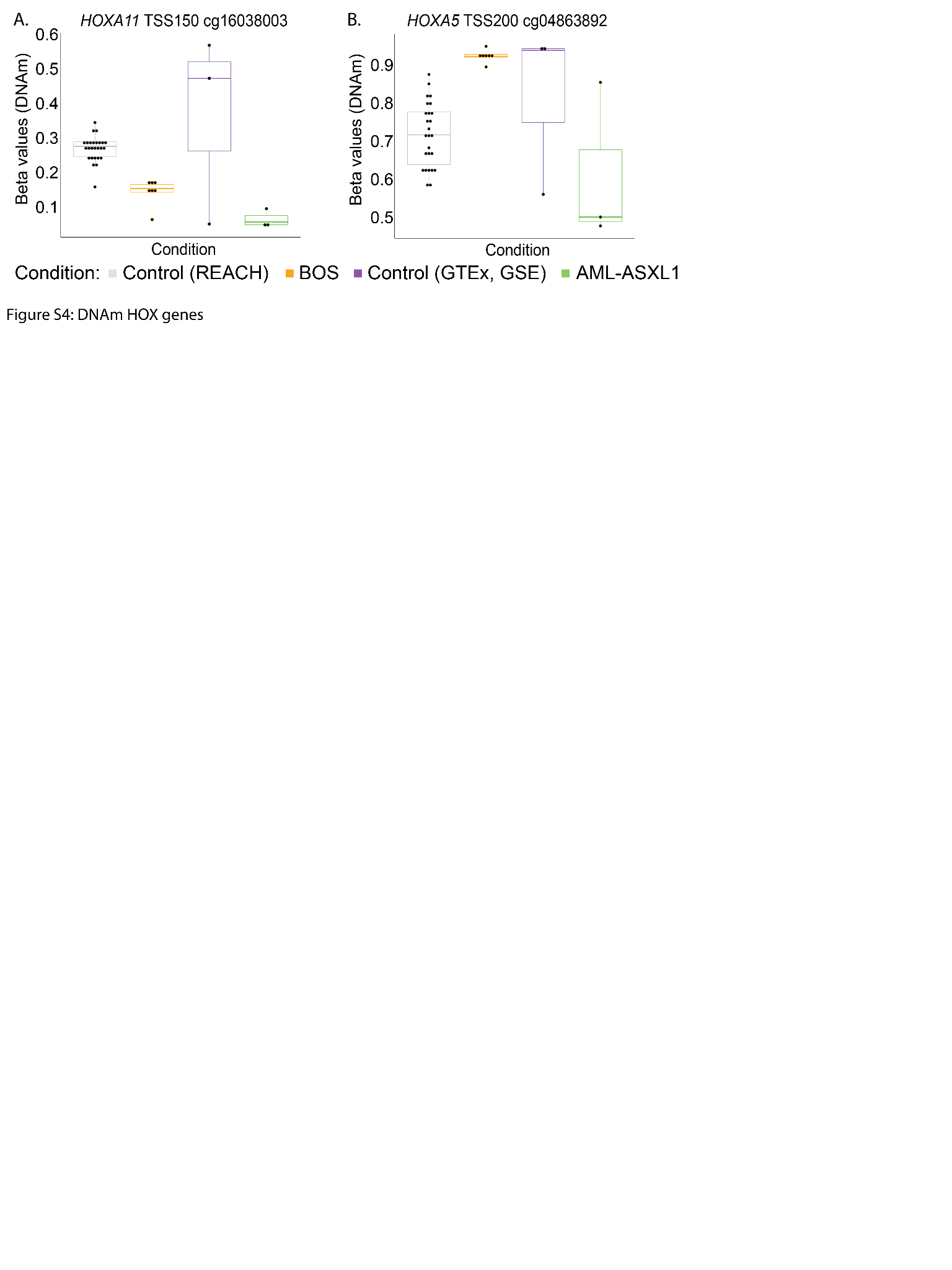


#### **Figure S4: DNAm of posterior *HOXA* transcriptional start site (TSS) CpG sites**

**(A)** DNAm β values for *HOXA11* TSS1500 at CpG site cg16038003 highlight the hypomethylation in both BOS and AML-ASXL1 patients compared to respective controls. **(B)** DNAm β values for *HOXA5* TSS200 at CpG site cg0486392 highlights the hypermethylation in BOS and hypomethylation in AML-ASXL1 patients compared to respective controls.


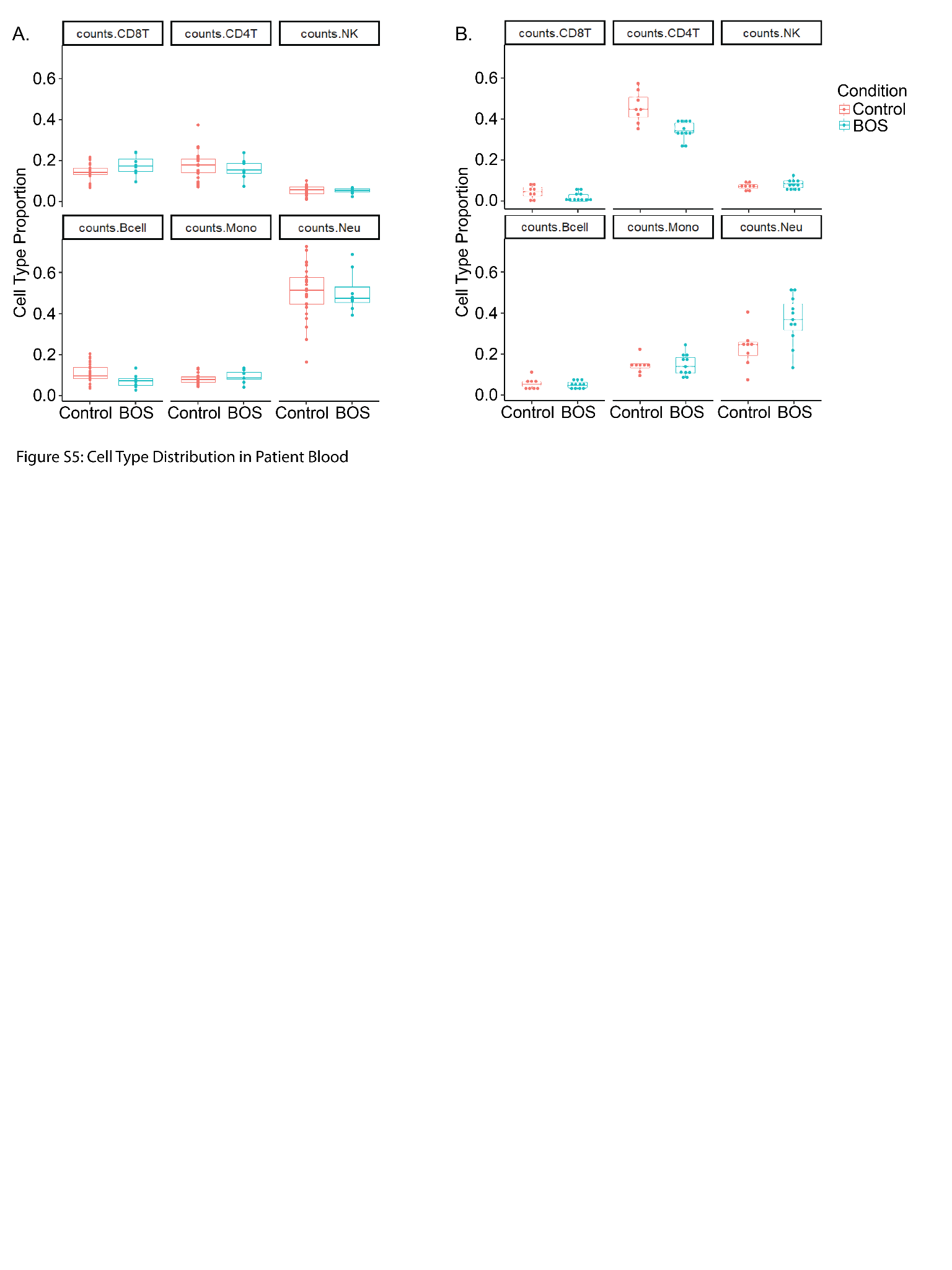


#### **Figure S5: Cell type distribution in blood samples reveals no significant differences in immune cell proportions in BOS.**

**(A)** Deconvolution of DNA methylation (DNAm) blood data, comparing the proportion of immune cell types between controls (red) and BOS samples (blue). The analysis includes CD8+ T cells, CD4+ T cells, NK cells, B cells, monocytes, and neutrophils. **(B)** Deconvolution of RNAseq blood data using *CIBERSORTx*, comparing the proportion of immune cell types between controls (red) and BOS samples (blue). The analysis includes CD8+ T cells, CD4+ T cells, NK cells, B cells, monocytes, and neutrophils.

#### **
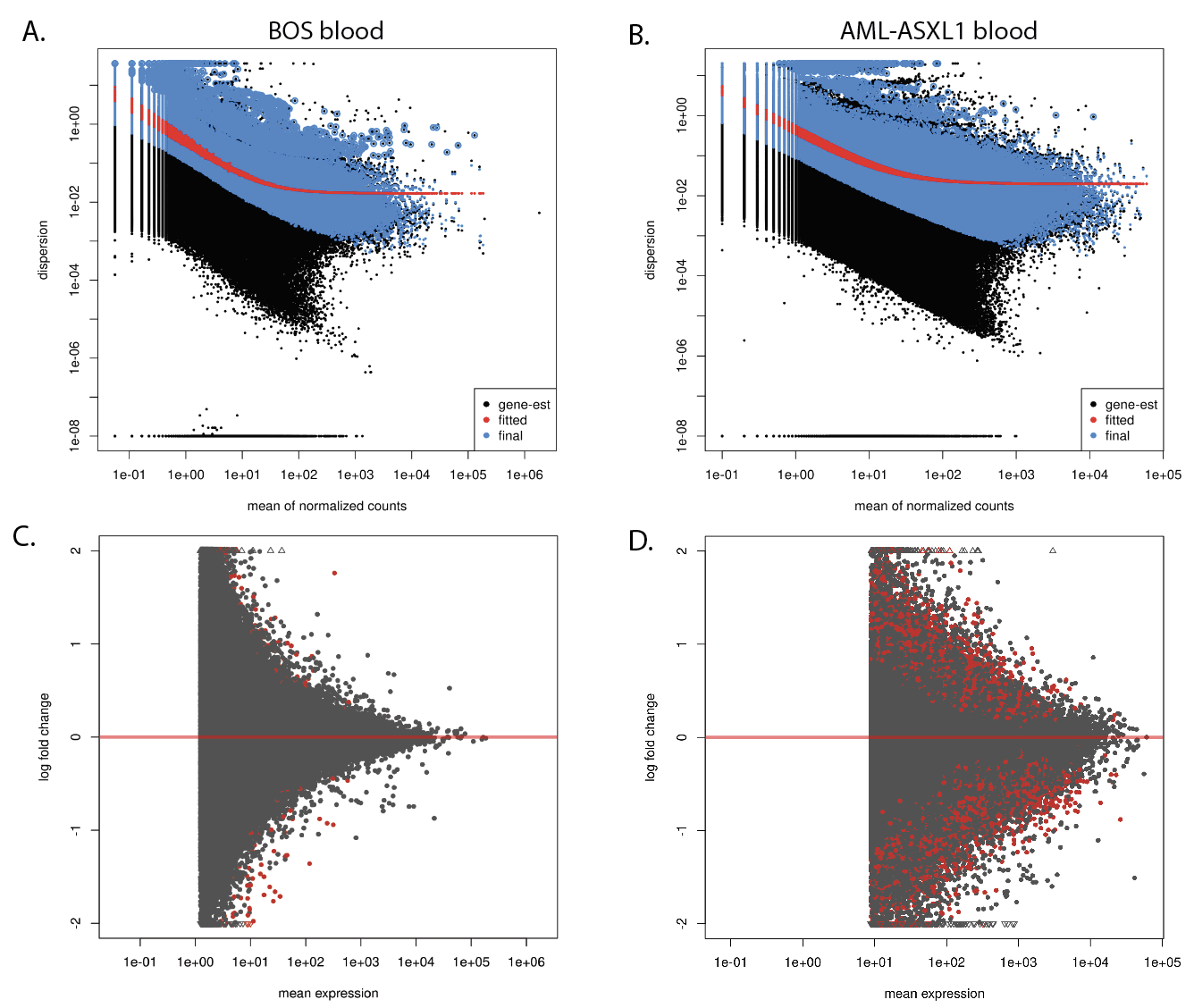
**

#### **Figure S6: DEXSeq quality control analysis**

A-B) Dispersion (per-exon dispersion estimates compared to mean normalized count) plot for (A) BOS blood and (B) AML-ASXL1 blood. Initial per-exon dispersion estimates are shown in black, fitted mean-dispersion function in red, and shrinked values in blue. C-D) MA plot (logarithm of fold change compared to mean normalized expression per exon) with significant hits at an FDR=0.05 shown in red for (C) BOS blood and (D) AML-ASXL1 blood.

**
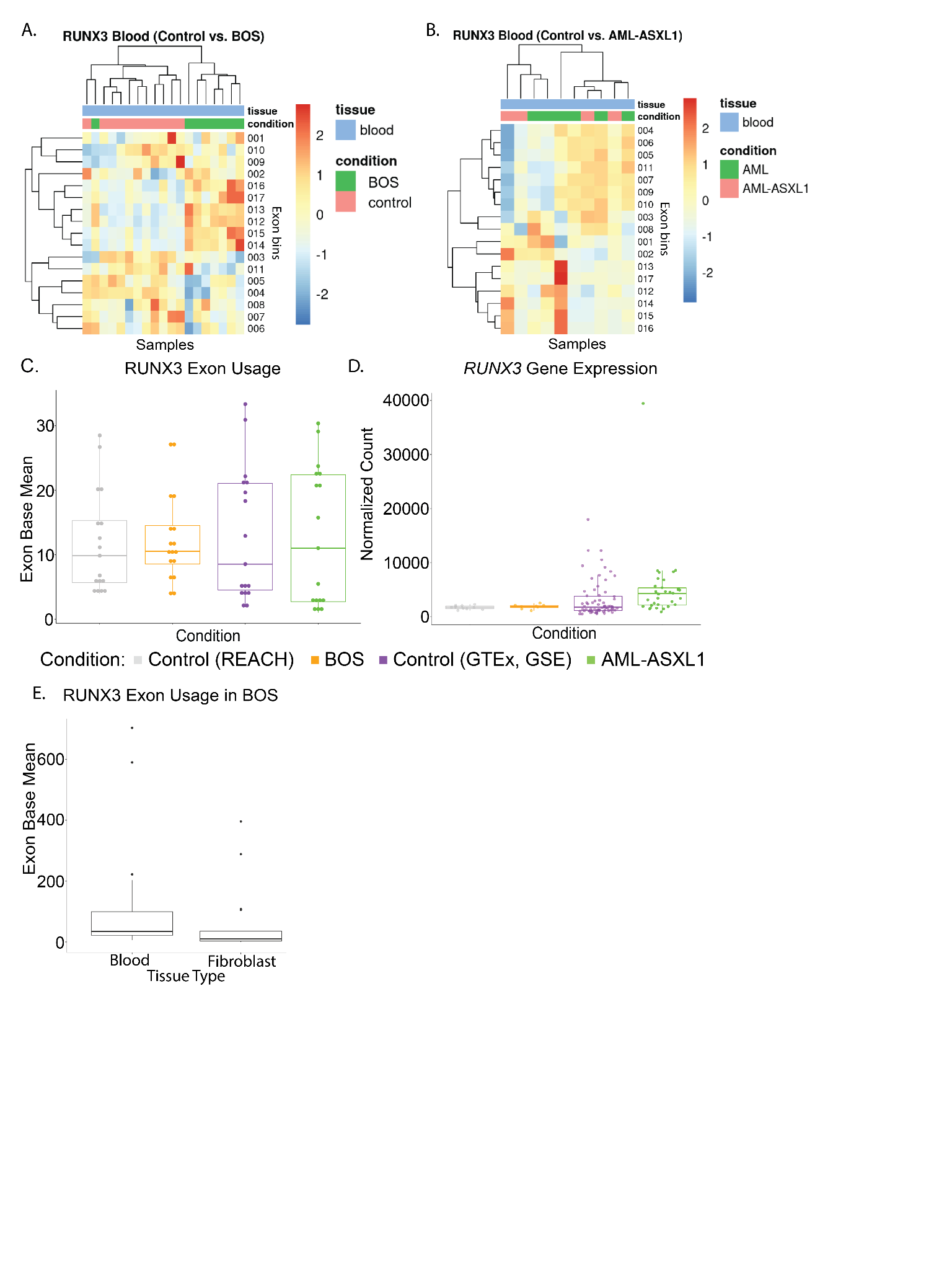
**

#### **Figure S7: *RUNX3* exon usage and gene expression**

A-B) Unsupervised hierarchical cluster heatmap of *RUNX3* exon bin usage in (A) BOS blood and (B) AML-ASXL1 blood. Cases are shown in green and controls in pink. C) Boxplot comparing mean exon usage of *RUNX3* in Controls, BOS, AML, and AML-ASXL1 (blood samples). D) Boxplot comparing normalized *RUNX3* gene expression in control blood, BOS blood, AML blood and bone marrow, and AML-ASXL1 blood and bone marrow. E) Boxplot comparing mean exon usage of *RUNX3* exon bins shows *RUNX3* is more highly expressed in BOS blood than fibroblast.

#### **
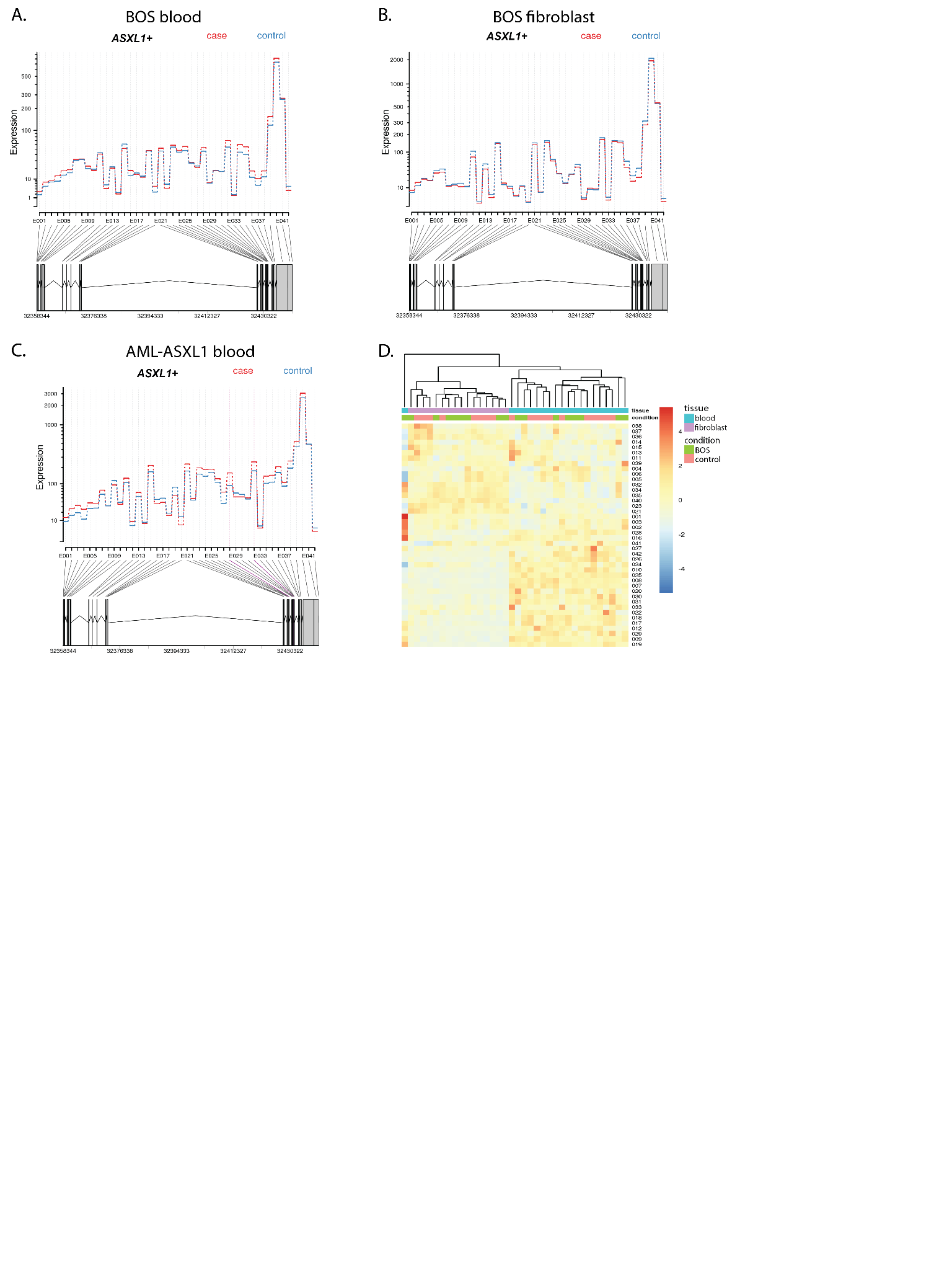
**

#### **Figure S8: *ASXL1* exon usage**

A-C) *ASXL1* fitted expression exon usage plots for (A) BOS blood, (B) BOS fibroblast, and (C) AML-ASXL1 blood. D) Unsupervised hierarchical cluster heatmap of *ASXL1* exon bin usage in BOS blood and fibroblast samples shows clustering by tissue type and not disease status (blue = blood, purple = fibroblast, green = case, pink = control).


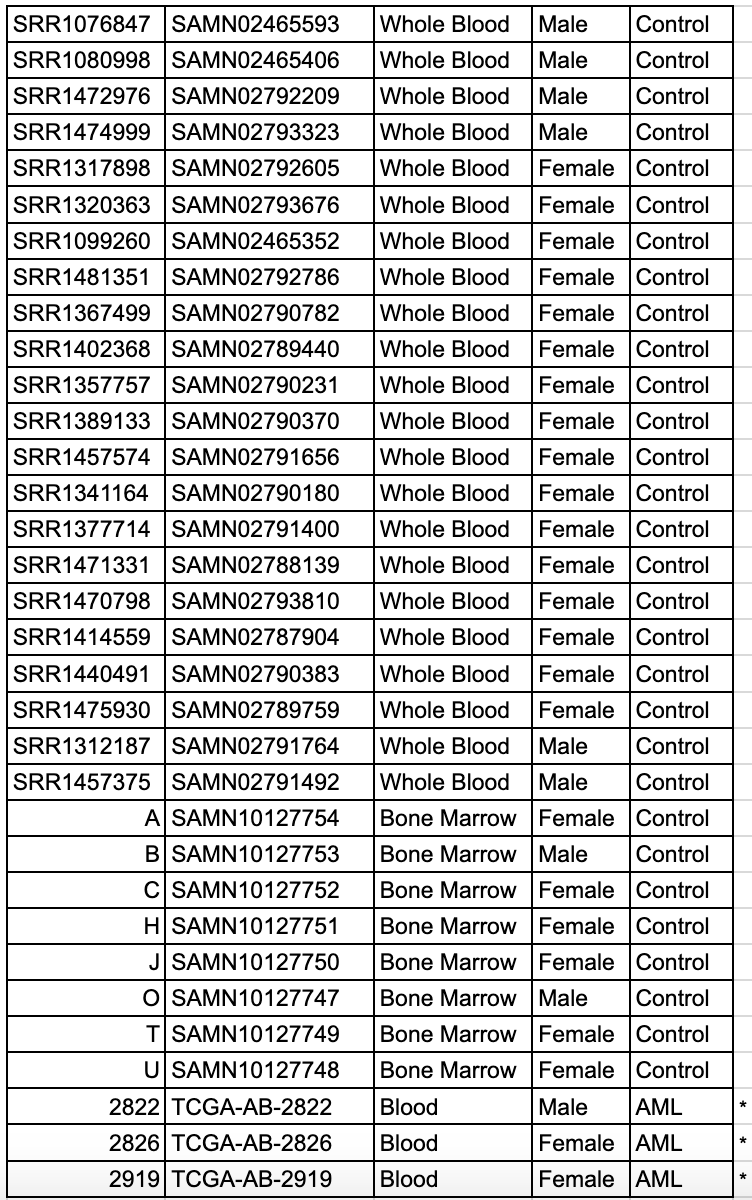


#### **Supplemental Table 1: Control sample characteristics for AML-ASXL1 analysis**

Demographic information for control samples used in the AML-ASXL1 analysis. This table includes sample ID, tissue type, and sex.


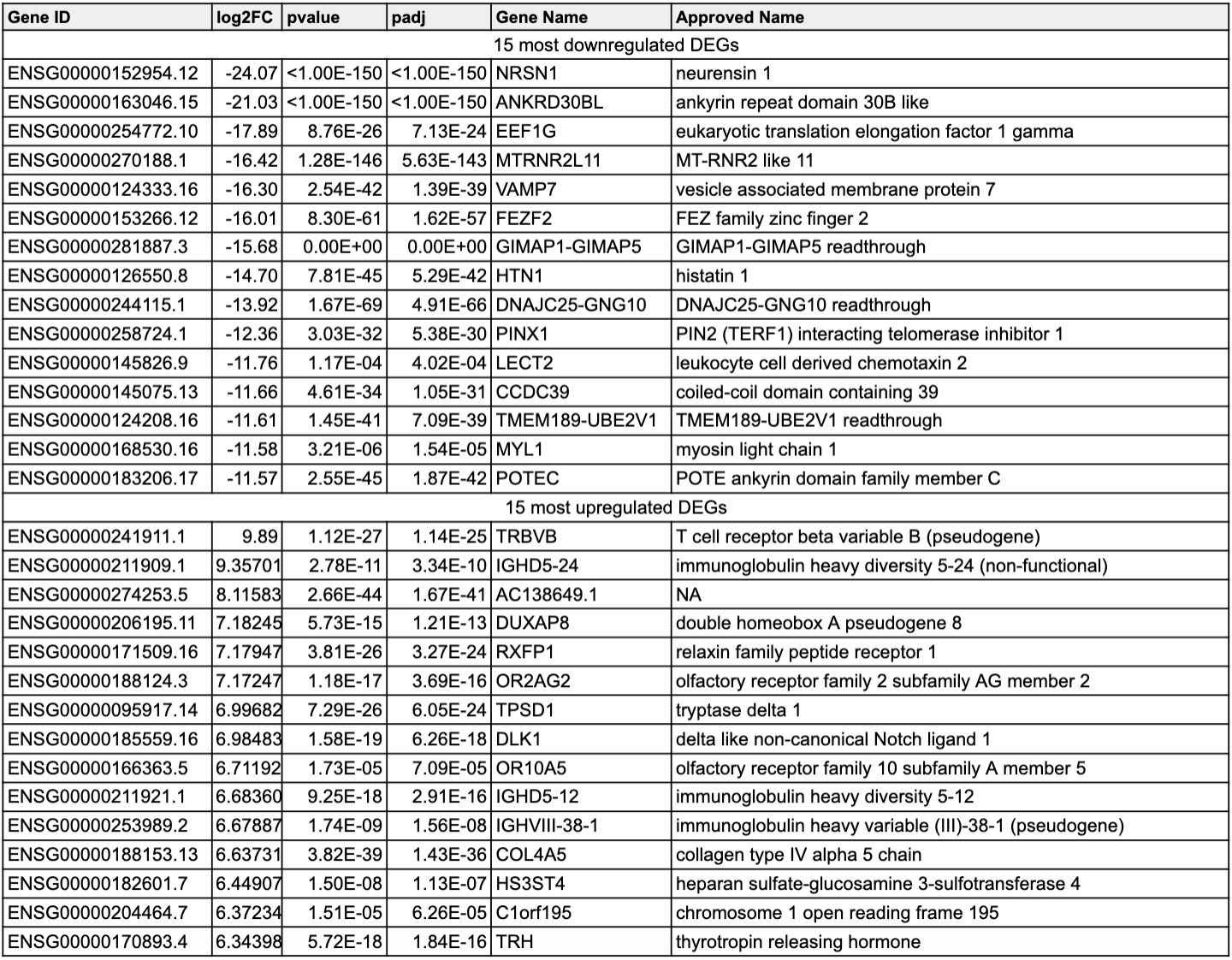


#### **Supplemental Table 2: Differential Gene Expression in AML-ASXL1 samples compared to controls (blood + bone marrow)**

This table shows the 15 most upregulated and 15 most downregulated differentially expressed genes (DEGs) with largest effect size from RNA sequencing comparing AML-ASXL1 samples and control samples from blood and bone marrow, adjusted for tissue and sex. The gene ID, log_2_ fold change (log_2_FC), p-value, adjusted p-value, and gene name are listed.


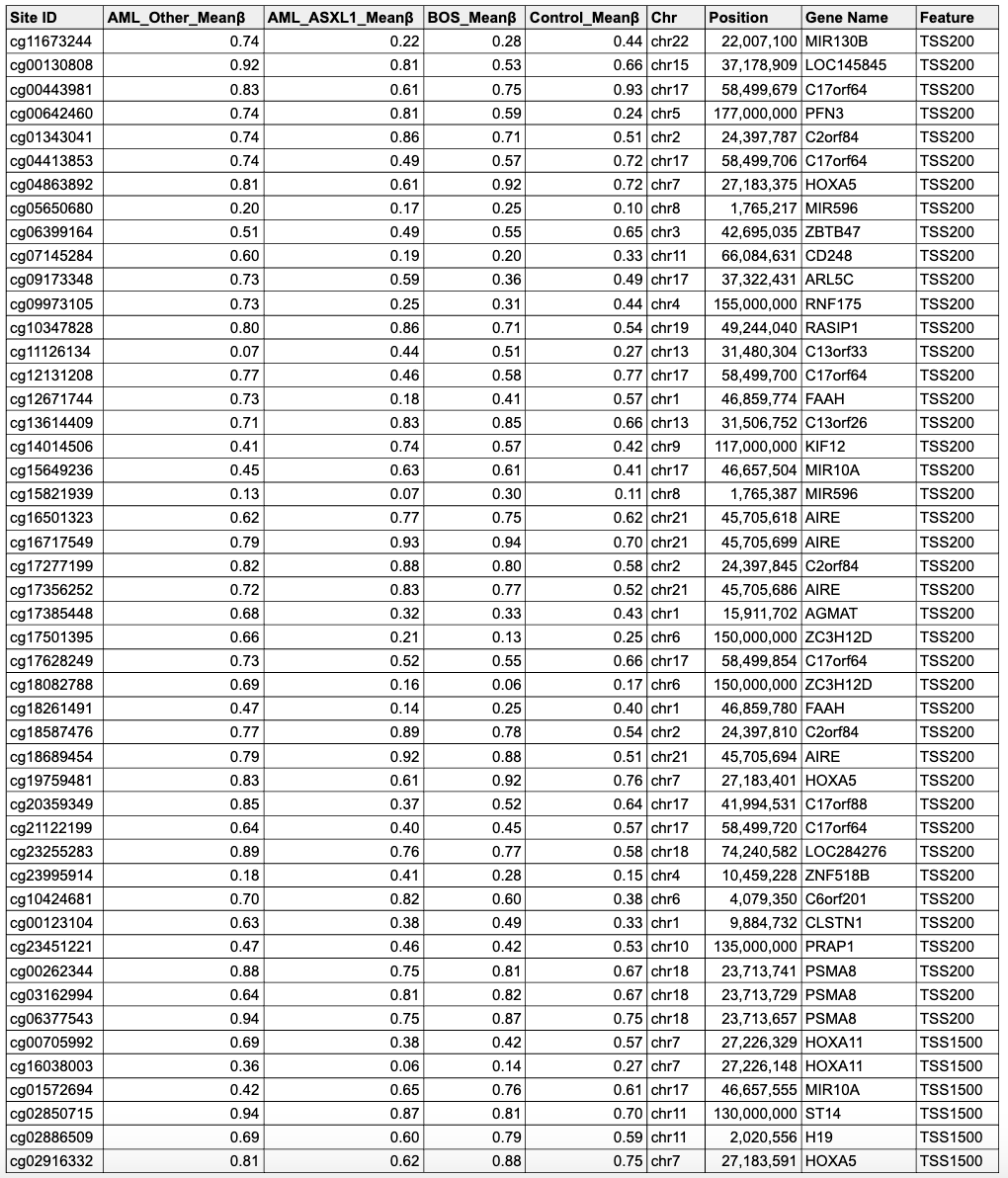


####
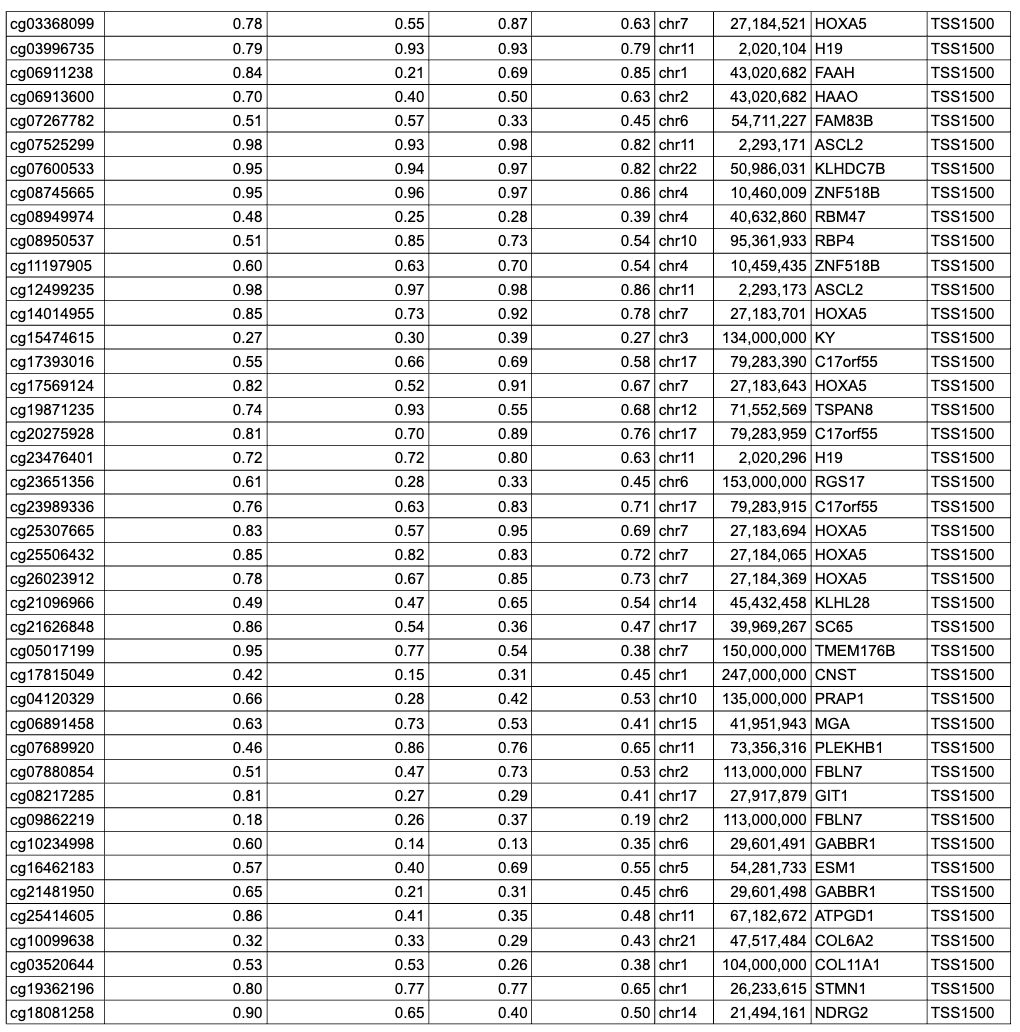
**Supplemental Table 3: DNA methylation at Transcriptional Start Sites (TSS).**

DNA methylation (DNAm) average mean DNAm beta (β) values at transcriptional start site (TSS) CpG sites, both 200bp upstream (TSS200) and 1500bp upstream (TSS1500), are shown. Samples analyzed are from patients with AML with non-ASXL1 mutations (AML-other, n=3), AML with *ASXL1* mutations (AML-*ASXL1*, n=3), Bohring-Opitz syndrome (BOS, n=8), and controls (n=26). The corresponding gene, CpG site, chromosome, and position for each CpG site are provided.


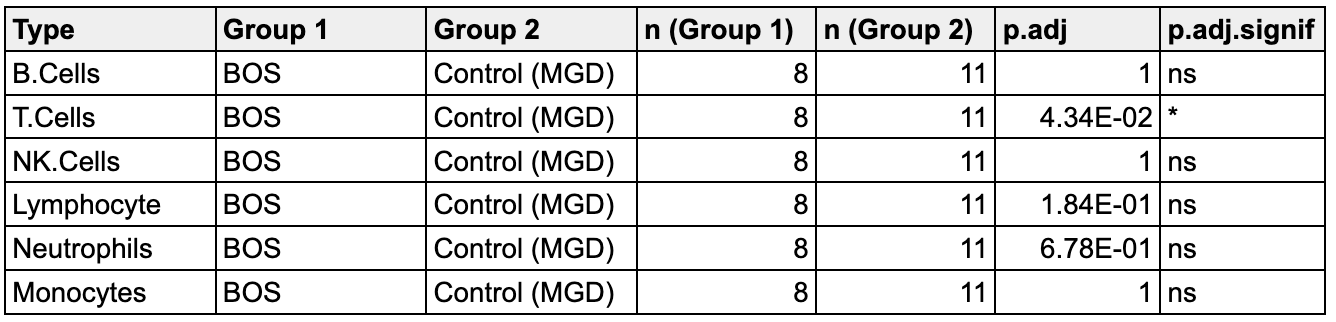


#### **Supplemental** Table 4: **CIBERSORTx cell type deconvolution in blood sample RNA sequencing**

This table summarizes the results from CIBERSORTx cell type deconvolution applied to RNA sequencing data from BOS and control blood samples.


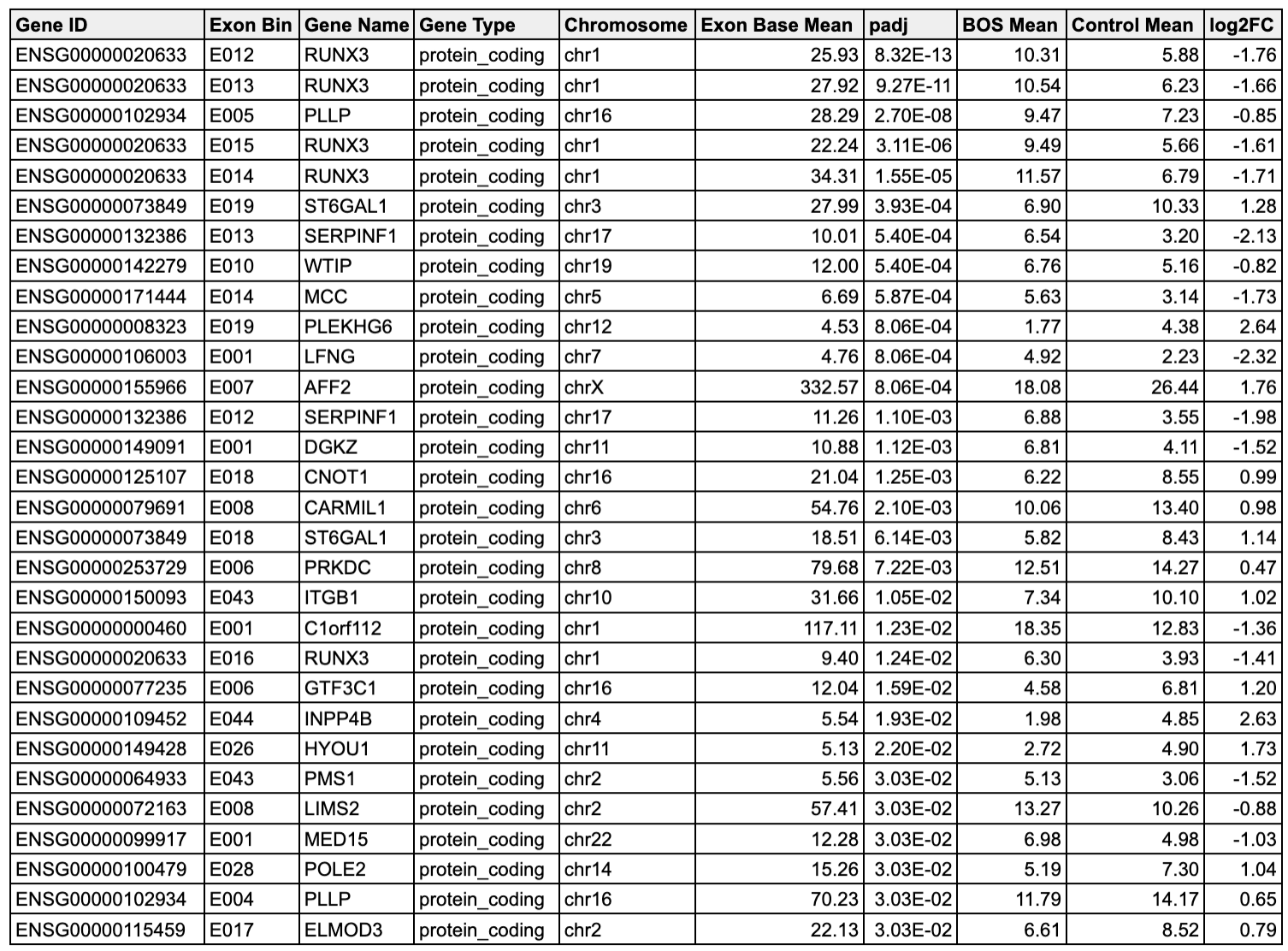


#### **Supplemental Table 5: Significant differential exon usage (DEU) exon bins in Bohring-Opitz syndrome blood samples.**

This table lists the most significant DEU exon bins identified in the comparison between Bohring-Opitz syndrome (BOS) samples and control samples. Each row represents an exon bin with detailed information on its statistical significance, including adjusted p-values and fold changes, highlighting the key exonic regions differentially used in BOS.

####


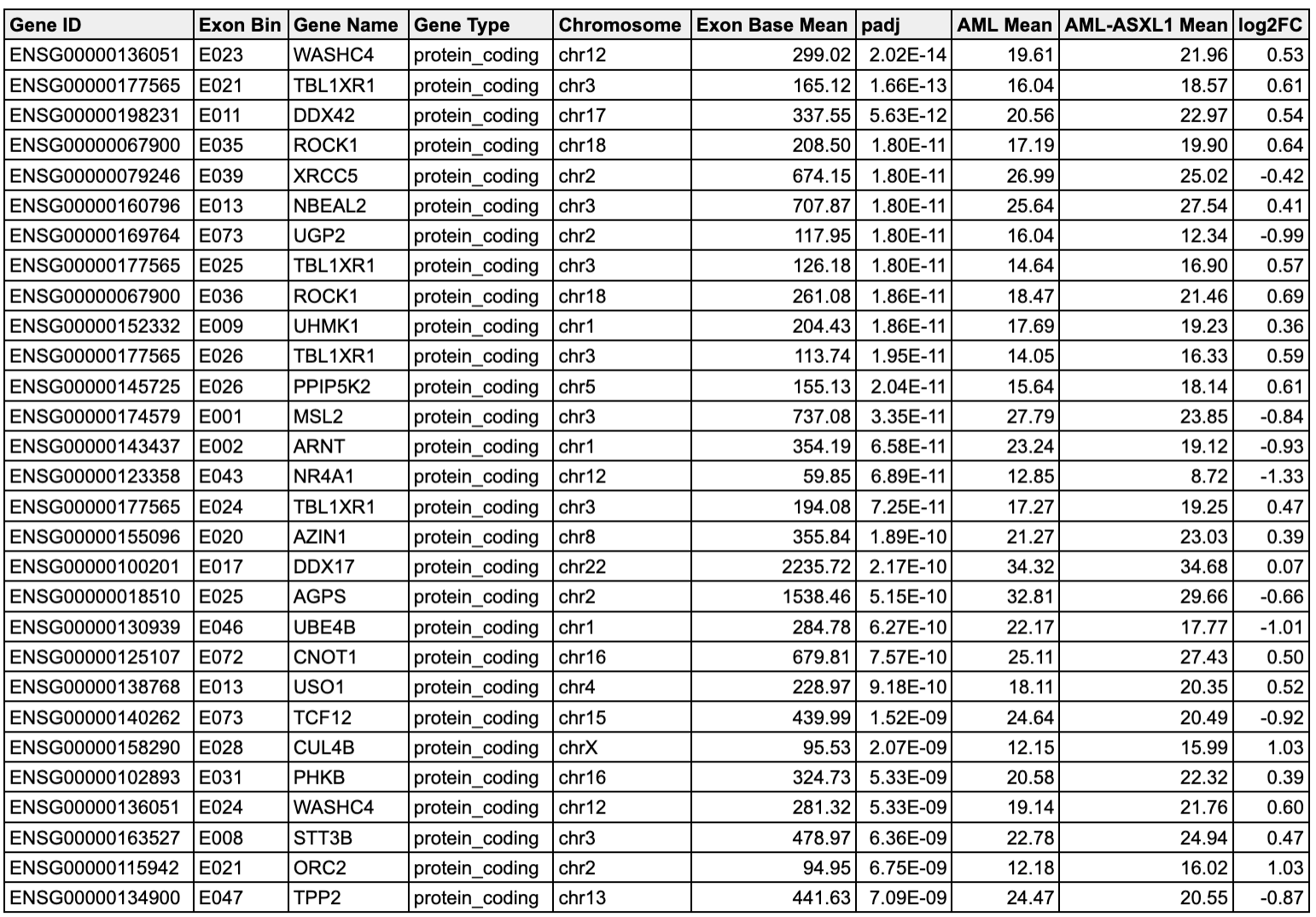


#### **Supplemental Table 6: Significant differential exon usage (DEU) exon bins in AML-*ASXL1* blood samples.**

This table presents the most significant DEU exon bins identified in the comparison between AML-*ASXL1* samples and control samples. The table provides comprehensive details on the differential usage of exon bins, including statistical significance metrics, which underscore the exonic regions with altered usage in AML-*ASXL1*.

####
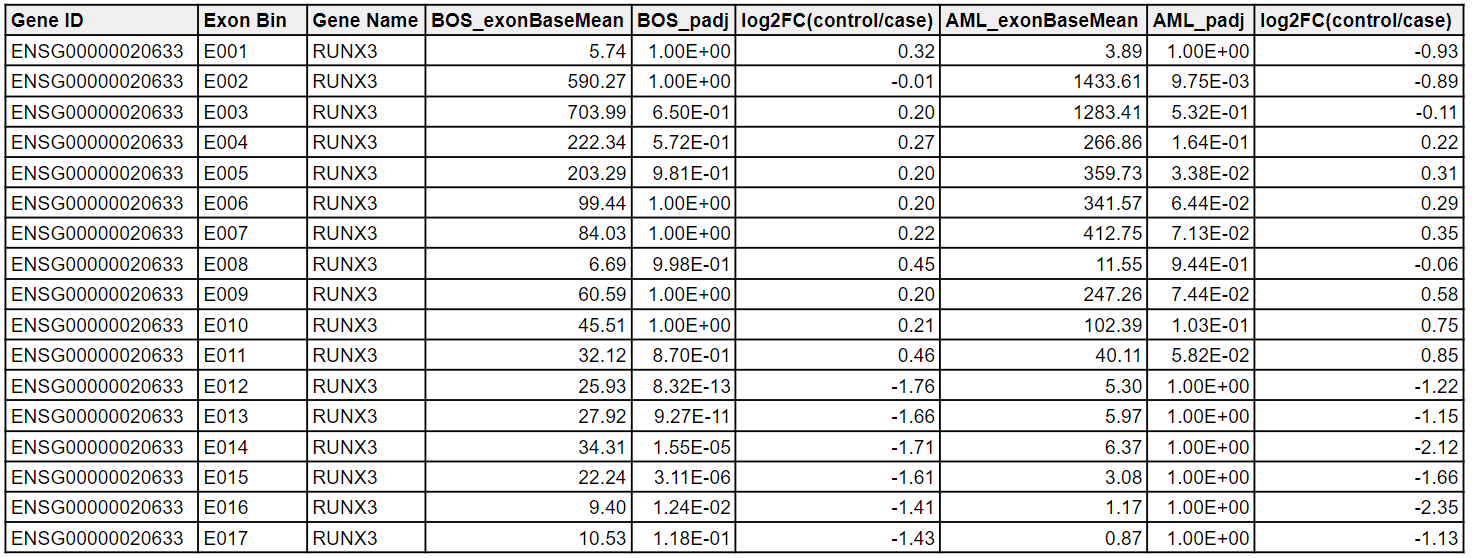


#### Supplemental Table 7: Differential exon usage in *RUNX3* across BOS and AML-*ASXL1*. The base means and log_2_ fold changes (log_2_FC) from differential exon usage analysis for each *RUNX3* exon bin are shown comparing BOS to controls and AML-*ASXL1* to controls.
